# Supplementary material for: Direct synthesis of sila-benzoazoles through hydrosilylation and rearrangement cascade reaction of benzoazoles and silanes
Source: Nat Commun. 2023 Feb 9;14:703. doi: 10.1038/s41467-023-36360-z (PMC9911738; doi:10.1038/s41467-023-36360-z)
Supplement: Supplementary file 1 — Supplementary Information [file 41467_2023_36360_MOESM1_ESM.docx]

Supplementary Information

Direct Synthesis of Sila-Benzoazoles through Hydrosilylation and Rearrangement Cascade Reaction of Benzoazoles and Silanes

Tianwei Liu, ^1,†^ Mo Yang, ^2,†^ Jianghua He,^1^ Shuhua Li,^2^,* and Yuetao Zhang^1^,*

^1^State Key Laboratory of Supramolecular Structure and Materials, College of Chemistry, Jilin University Changchun, Jilin, 130012 (China)

^2^Key Laboratory of Mesoscopic Chemistry of Ministry of Education, Institute of Theoretical and Computational Chemistry, School of Chemistry and Chemical Engineering, Nanjing University, Nanjing, 210093 (China)

^†^These authors contributed equally.

^*^E-mail: [ytzhang2009@jlu.edu.cn](mailto:ytzhang2009@jlu.edu.cn); [shuhua@nju.edu.cn](mailto:shuhua@nju.edu.cn)

Content

[1. Materials, reagents and methods 3](#_Toc125275406)

[1.1 Preparation of starting materials 3](#_Toc125275407)

[1.2 General procedure for silane insertion reaction 8](#_Toc125275408)

[2. Gram-scale reaction 9](#_Toc125275409)

[3. NMR data for product 10](#_Toc125275410)

[4. Supplementary Table 1. Optimization of silane insertion reaction 19](#_Toc125275411)

[5. Comparison of the atomic efficiency of our strategy with that of traditional method 20](#_Toc125275412)

[6. Mechanism studies 21](#_Toc125275413)

[6.1 In-situ monitoring of the silane insertion reaction 21](#_Toc125275414)

[6.2 Reaction of B(C_6_F_5_)_3_ and 1a 22](#_Toc125275415)

[6.3 Deuterium-labelled NMR reaction 24](#_Toc125275416)

[6.4 Using tertiary silane as silicon source 25](#_Toc125275417)

[6.5 Gibbs free energy profile for the possible hydrosilylation and ring-closing/ring-opening pathway 26](#_Toc125275418)

[6.6 In-situ NMR reaction heating at 80ºC or 110ºC 26](#_Toc125275419)

[6.7 Reaction of Int5-Me with B(C_6_F_5_)_3_ 27](#_Toc125275420)

[7. X-ray single crystal data 28](#_Toc125275421)

[8. DFT calculations 30](#_Toc125275422)

[9. NMR spectra 32](#_Toc125275423)

[10. References 101](#_Toc125275424)

1. **Materials, reagents and methods**

All the synthesis and manipulations of air- and moisture-sensitive materials were carried out in flamed Schlenk-type glassware on a dual-manifold Schlenk line, or an argon-filled glovebox. Hexane, THF and Et_2_O were refluxed over CaH_2_, followed by distillation under nitrogen atmosphere, then stored over molecular sieves 4 Å. CDCl_3_, CHCl_3_, C_6_H_6_ and C_6_D_6_ were dried over molecular sieves 4 Å. NMR spectra were recorded on a Bruker Avance II 500 (500 MHz, ^1^H; 126 MHz, ^13^C; 471 MHz, ^19^F; 99 MHz, ^29^Si) or Zhongke Niujin AS 400 (400 MHz, ^1^H) instrument at room temperature (RT). Chemical shifts for ^1^H and ^13^C NMR spectra were referenced to internal solvent resonances and reported as parts per million relatively to SiMe_4_, whereas ^19^F NMR spectra were referenced to external CFCl_3_. Air sensitive NMR samples were conducted in Teflon-valve sealed J. Young-type NMR tubes. High Resolution Mass Spectra (HRMS) were recorded on the Bruker MicroTOF Q II. It should be noted that the new sila-benzoazoles reported in the manuscript were characterized by ^1^H, ^13^C and ^29^Si NMR spectroscopy without HRMS or elemental analyses, due to their extreme sensitivity to air and moisture.

Benzothiazoles including **1a** to **1e**, **1m** to **1p** and **1u** to **1w**, benzoxazoles including **2a** to **2k,** silanes including Ph_2_SiH_2_, Et_2_SiH_2_, MePhSiH_2_ were purchased from energy chemical (Shanghai, China). Substrates **1j** and PhSiH_3_ were purchased from J&K (Beijing, China). Et_3_N was purchased from Titan (Shanghai, China). Unless otherwise specified, and all chemicals were used as received unless otherwise specified. B(C_6_F_5_)_3_ was prepared according to literature procedures.^1^ Benzoselazoles **3a** and **3b** were prepared according to literature procedures.^2, 3^ Benzothiazoles **1f** to **1i**, **1k**, **1l**, **1q to 1t** and 4,4'-disilyl-1,1'-biphenyl (**e**) as well were prepared according to literature procedures (Check section 1.1 for details).^4-6^

## Preparation of starting materials

**Synthesis of B(C_6_F_5_)_3_**

*^n^*BuLi (2.5 M, 72 mL, 0.18 mol) was added dropwise to a solution of C_6_F_5_Br (44.5 g, 0.18 mol) in dry hexane (400 mL) at -78ºC under N_2_ atmosphere over a period of 15 minutes. BCl_3_ (1.0 M, 60 mL, 0.06 mol) was quickly added to the above mixture, and then slowly warmed to RT and reaction for another 4 h. After the reaction, the solvent was removed by vacuum, the first sublimation temperature was 80ºC to remove yellow oily by-product, and the second sublimation temperature was 120ºC to obtain pure product.

B(C_6_F_5_)_3_, White solid (40%) ^19^F NMR (471 MHz, CDCl_3_, 25ºC) δ -127.8, -142.7, -159.9. (Known compound, prepared according to literature procedures. Spectroscopic data was consistent with literature)^1^

**Synthesis of benzoselazoles** **3a**

An oven-dried 500 mL pressure tube were added with 2-iodoaniline (3.3 g, 15 mmol, 1 equiv), Se (3.6 g 45 mmol, 3 equiv), CuBr (216 mg, 1.5 mmol, 10 mol %), KOH (1.7 g, 30 mmol, 2 equiv), DMSO (150 mL). The tube was degassed to remove oxygen and filled with nitrogen. The reaction mixture was stirred at 120ºC for 8 h. After cooled down to RT, the reaction mixture was poured into saturated Na_2_CO_3_ solution (150 mL) and extracted with EA. The combined organic phase was dried over Na_2_SO_4_, filtered and concentrated in vacuo. The residue was purified by column chromatography on silica gel to afford the 2,2'-diselanediyldianiline. An oven-dried 200 mL reaction bottle were added with 2,2'-diselanediyldianiline (2.1 g, 6 mmol, 1 equiv), Tol (80 mL), then Bu_3_P (3.6 g, 18 mmol) was added to the above mixture for 5 min under nitrogen atmosphere at RT. HCOOH (0.28 g, 6 mmol) was added to the above mixture and reaction at 110ºC for 48 h. After cooled down to RT, the reaction mixture was poured into saturated Na_2_CO_3_ solution (80 mL) and extracted with DCM. The combined organic phase was dried over MgSO_4_, filtered and concentrated in vacuo. The residue was purified by column chromatography on silica gel to afford product **3a**.

benzo[d][1,3]selenazole (**3a**) colorless oil (40%) ^1^H NMR (400 MHz, CDCl_3_) δ 9.94 (s, 1H, HAr), 8.19 (d, *J* = 8.0 Hz, 1H, HAr), 8.00 (d, *J* = 8.0 Hz, 1H, HAr), 7.50 (t, *J* = 8.4 Hz, *J* = 1.0 Hz, 1H, HAr), 7.36 (t, *J* = 8.4 Hz, *J* = 1.0 Hz, 1H, HAr). (Known compound, prepared according to literature procedures. Spectroscopic data was consistent with literature)^2,3^

**Synthesis of benzoselazoles** **3b**

An oven-dried 200 mL Schlenk bottle were added with 2-iodoaniline (3.3 g, 15 mmol, 1 equiv), THF (100 mL) under a nitrogen atmosphere at -78ºC, then nBuLi (18 mL, 2.5M in Hex) was added within 15 min, the resulting mixture was stirred at -78ºC for 1 h. Selenium powder was added to the above reaction mixture and then warm to RT for 1 h, after which it was diluted with a saturated solution of NH_4_Cl and stirred in flowing air for 2 h. It was extracted with EA. The combined organic phase was dried over Na_2_SO_4_, filtered and concentrated in vacuo. The residue was purified by column chromatography on silica gel to afford the 2,2'-diselanediyldianiline. An oven-dried 200 mL reaction bottle were added with 2,2'-diselanediyldianiline (2.2 g, 6.3 mmol, 1 equiv), Tol (80 mL), then Bu_3_P (3.8 g, 18.9 mmol) was added to the above mixture for 5 min under nitrogen atmosphere at RT. CH_3_COOH (0.38 g, 6.3 mmol) was added to the above mixture and reaction at 110ºC for 12 h. After cooled down to RT, the reaction mixture was poured into saturated Na_2_CO_3_ solution (80 mL) and extracted with DCM. The combined organic phase was dried over MgSO_4_, filtered and concentrated in vacuo. The residue was purified by column chromatography on silica gel to afford product **3b**.

2-methylbenzo[d][1,3]selenazole (**3b**) Light yellow oil (52%) ^1^H NMR (400 MHz, CDCl_3_) δ 7.98 (d, *J* = 8.0 Hz, 1H, HAr), 7.85 (d, *J* = 8.0 Hz, 1H, HAr), 7.42 (t, *J* = 8.0 Hz, *J* = 1.0 Hz, 1H, HAr), 7.26 (t, *J* = 8.0 Hz, *J* = 1.0 Hz, 1H, HAr). 2.87 (s, 3H, CH_3_), (Known compound, prepared according to literature procedures. Spectroscopic data was consistent with literature)^2,3^

**Synthesis of benzothiazoles 1f to 1h and 1l**

An oven-dried 20 mL pressure tube were added with 5-bromobenzothiazole (428 mg, 2 mmol, 1 equiv), an arylboronic acid (3 mmol, 1.5 equiv), Pd(PPh_3_)_4_ (115.6 mg, 0.1 mmol, 5 mol %), K_2_CO_3_ (1.1 g, 8 mmol, 4 equiv), toluene (10 mL) and H_2_O (2 mL). The tube was degassed to remove oxygen and filled with nitrogen. The reaction mixture was stirred at 80ºC for 8 h. After cooled down to room temperature, the reaction mixture was poured into water (8 mL). The organic phase was collected and water phase was washed by dichloromethane (10 mL × 3). The combined organic phase was dried over Na_2_SO_4_, filtered and concentrated in vacuo. The residue was purified by column chromatography on silica gel to afford the corresponding products.

5-(thiophen-2-yl)benzo[d]thiazole (**1f**) White solid (30%) ^1^H NMR (500 MHz, CDCl_3_) δ 9.04 (s, 1H, HAr), 8.40 (s, 1H, HAr), 7.92 (d, *J* = 8.5 Hz, 1H, HAr), 7.70 (dd, *J_1_* = 8.5 Hz, *J_2_* = 1.0 Hz, 1H, HAr), 7.42 (d, *J* = 3.5 Hz, 1H, HAr), 7.33 (d, *J* = 5.0 Hz, 1H, HAr), 7.12 (dd, *J_1_* = 5.0 Hz, *J_2_* = 4.0 Hz, 1H, HAr), ^13^C NMR (126 MHz, CDCl_3_) δ 154.9, 154.0, 143.7, 133.0, 132.8, 128.2, 125.3, 124.0, 123.7, 122.2, 120.5. HRMS (ESI) m/z calcd for C_11_H_8_NS_2_ [M + H]^+^ : 218.0093, found: 218.0094.

5-(benzo[b]thiophen-2-yl)benzo[d]thiazole (**1g**) White solid (30%) ^1^H NMR (500 MHz, CDCl_3_) δ 9.06 (s, 1H, HAr), 8.50 (s, 1H, HAr), 7.97 (d, *J* = 8.0 Hz, 1H, HAr), 7.86 (d, *J* = 7.5 Hz, 1H, HAr), 7.81 (d, *J* = 8.0 Hz, 2H, HAr), 7.65 (s, 1H, HAr), 7.34 – 7.41 (m, 2H, HAr). ^13^C NMR (126 MHz, CDCl_3_) δ 155.0, 153.8, 143.5, 140.7, 139.6, 133.4, 132.9, 124.7, 124.6, 124.2, 123.7, 122.3, 122.2, 121.2, 120.1. HRMS (ESI) m/z calcd for C_15_H_10_NS_2_ [M + H]^+^ : 268.0249, found: 268.0254.

5-([1,1'-biphenyl]-4-yl)benzo[d]thiazole (**1h**) Yellow solid (80%) ^1^H NMR (500 MHz, CDCl_3_) δ 9.08 (s, 1H, HAr), 8.46 (s, 1H, HAr), 8.04 (d, *J* = 8.5 Hz, 1H, HAr), 7.70 – 7.82 (m, 7H, HAr), 7.50 (t, *J* = 7.0 Hz, 2H, HAr), 7.40 (t, *J* = 7.5 Hz, 1H, HAr). ^13^C NMR (126 MHz, CDCl_3_) δ 154.7, 154.1, 140.6, 140.5, 139.4, 139.4, 132.8, 128.9(2C), 127.8(2C), 127.7(2C), 127.5, 127.1(2C), 125.0, 122.1, 121.8. HRMS (ESI) m/z calcd for C_19_H_14_NS [M + H]^+^ : 288.0841, found: 288.0837.

5-(benzo[b]thiophen-2-yl)benzo[d]thiazole (**1l**) White solid (10%) ^1^H NMR (500 MHz, CDCl_3_) δ 9.06 (s, 1H, HAr), 8.95 (d, *J* = 2.0 Hz, 1H, HAr), 8.63 (dd, *J_1_* = 4.5 Hz, *J_2_* = 1.0 Hz, 1H, HAr), 8.35 (d, *J* = 1.0 Hz, 1H, HAr), 8.05 (d, *J* = 8.5 Hz, 1H, HAr), 7.95 (dt, *J_1_* = 7.5 Hz, *J_2_* = 1.5 Hz, 1H, HAr), 7.66 (dd, *J_1_* = 8.5 Hz, *J_2_* = 2.0 Hz, 1H, HAr), 7.40 (dd, *J_1_* = 7.5 Hz, *J_2_* = 4.5 Hz, 1H, HAr). ^13^C NMR (126 MHz, CDCl_3_) δ 155.0, 154.0, 148.5, 136.1, 134.7, 133.6, 132.0, 128.5, 124.8, 123.7, 122.5, 122.0. HRMS (ESI) m/z calcd for C_12_H_9_N_2_S [M + H]^+^ : 213.0481, found: 213.0474

**Synthesis of benzothiazole 1i and 1k**

An oven-dried 20 mL pressure tube were added with 6-bromobenzothiazole (428 mg, 2 mmol, 1 equiv), an arylboronic acid (3 mmol, 1.5 equiv), Pd(PPh_3_)_4_ (115.6 mg, 0.1 mmol, 5 mol %), K_2_CO_3_ (1.1 g, 8 mmol, 4 equiv), toluene (10 mL) and H_2_O (2 mL). The tube was degassed to remove oxygen and filled with nitrogen. The reaction mixture was stirred at 80ºC for 8 h. After cooled down to room temperature, the mixture was poured into water (8 mL). The organic phase was collected and water phase was washed by dichloromethane (10 mL × 3). The combined organic phase was dried over Na_2_SO_4_, filtered and concentrated in vacuo. The residue was purified by column chromatography on silica gel to afford the corresponding products.

1. (naphthalen-1-yl)benzo[d]thiazole (**1i**) Yellow solid (70%) ^1^H NMR (500 MHz, CDCl_3_) δ 9.09 (s, 1H, HAr), 8.29 (d, *J* = 8.5 Hz, 1H, HAr), 8.09 (s, 1H, HAr), 7.91 – 7.98 (m, 3H, HAr), 7.69 (dd, *J_1_* = 8.0 Hz, *J_2_* = 1.0 Hz, 1H, HAr), 7.46 – 7.60 (m, 4H, HAr). ^13^C NMR (126 MHz, CDCl_3_) δ 154.3, 152.5, 139.3, 138.5, 134.0, 133.8, 131.7, 128.8, 128.4, 128.1, 127.4, 126.4, 126.0, 125.8, 125.4, 123.2, 123.0. HRMS (ESI) m/z calcd for C_17_H_12_NS [M + H]^+^ : 262.0685, found: 262.0692.

6-(3,5-bis(trifluoromethyl)phenyl)benzo[d]thiazole (**1k**) White solid (50%) ^1^H NMR (500 MHz, CDCl_3_) δ 9.08 (s, 1H, HAr), 8.25 (d, *J* = 8.5 Hz, 1H, HAr), 8.20 (d, *J* = 1.5 Hz, 1H, HAr), 8.09 (s, 2H, HAr), 7.92 (s, 1H, HAr), 7.75 (dd, *J_1_* = 8.5 Hz, *J_2_* = 2.0 Hz, 1H, HAr). ^13^C NMR (126 MHz, CDCl_3_) δ 155.2, 153.5, 142.6, 135.9, 134.9, 132.7, 132.5, 132.2, 131.9, 127.5, 125.7, 124.3, 122.2, 121.2, 120.6. HRMS (ESI) m/z calcd for C_17_H_12_NS [M + H]^+^ : 348.0276, found: 348.0274.

**Synthesis of Int5-Me**

In a glove box, 2-(ethylideneamino)benzenethiol^7^ (30.2 mg, 0.2 mmol) and Ph_2_SiClH (43.6 mg, 0.2 mmol)^8^ were added to a 10 mL Schlenk bottle containing 1 mL dry benzene. Taking the bottle out of glove box, Et_3_N (202 mg, 2 mmol) was added to the mixture under N_2_ atmosphere and then heated at 80ºC for 24 h. After reaction, solvent and excess Et_3_N were removed under the vacuum, then washed by hexane for three times. The supernatant was collected and put in a refrigerator at -35ºC for 24 h, then collected the up-layer and dried in vacuo to afford the final product.^9^

N-(2-((diphenylsilyl)thio)phenyl)ethanimine (**Int5-Me**) viscous liquid (85%) ^1^H NMR (500 MHz, CDCl_3_) δ 7.72 – 7.73 (m, 4H, HAr), 7.46 – 7.55 (m, 6H, HAr), 7.20 (dd, *J_1_* = 7.5 Hz, *J_2_* = 1.0 Hz, 1H, HAr), 6.85 (td, *J_1_* = 8.0 Hz, *J_2_* = 1.5 Hz, 1H, HAr), 6.78 (td, *J_1_* = 7.5 Hz, *J_2_* = 0.5 Hz, 1H, HAr), 6.65 (d, *J* = 8.0 Hz, 1H, HAr), 5.68 (s, 1H, SiH), 5.23 – 5.26 (m, 1H, CH), 1.49 (d, *J* = 6.0 Hz, 3H, CH_3_). ^13^C NMR (126 MHz, CDCl_3_) δ 145.8, 135.5(2C), 135.3(2C), 131.9, 131.5, 130.8, 128.9, 128.5, 128.35(2C), 128.41(2C), 124.9, 122.3, 120.4, 112.0, 65.7, 28.0.

## 1.2 General procedure for silane insertion reaction

**Typical procedure for optimization**

In a glovebox, benzoazole (0.1 mmol) and catalyst (0.005 mmol) were dissolved in CDCl_3_ (0.6 mL) in a 2-mL NMR tube, then Ph_2_SiH_2_ (18.4 mg, 0.1 mmol) and mesitylene (12.0 mg, 0.1 mmol) were added to the above mixture. Reaction was performed under the specific conditions, then measured by ^1^H NMR spectroscopy.

**Typical procedure for synthesis of 6fa**, **6ga**, **6ma-6qa**, **6sa-6ua**, **6ac**, **6ad, 7aa-7ca, 7ea-7ka**, **7ac**, **7ad**, **8aa**, **8ba**, **8ac**, **8ad**

In a glovebox, benzoazole (0.1 mmol) and B(C_6_F_5_)_3_ (2.6 mg, 0.005 mmol) were dissolved in CDCl_3_ (0.6 mL) in a 2-mL NMR tube, then corresponding silane (0.1 mmol) and mesitylene (12.0 mg, 0.1 mmol) were added to the above mixture. The reaction was heated at 110ºC for 8 h, then measured by ^1^H NMR spectroscopy.

**Typical procedure for synthesis of 6ba** **and 6ea**

In a glovebox, benzoazole (0.1 mmol) and B(C_6_F_5_)_3_ (2.6 mg, 0.005 mmol) were dissolved in CDCl_3_ (0.6 mL) in a 2-mL NMR tube, then Ph_2_SiH_2_ (0.1 mmol) and mesitylene (12.0 mg, 0.1 mmol) were added to the above mixture. The reaction was heated at 110ºC for 24 h, then measured by ^1^H NMR spectroscopy.

**For synthesis of** **6ja**

In a glovebox, benzoazole (0.2 mmol) and B(C_6_F_5_)_3_ (5.1 mg, 0.01 mmol) were dissolved in CDCl_3_ (0.2 mL) in a 2-mL NMR tube, then Ph_2_SiH_2_ (0.2 mmol) and mesitylene (12.0 mg, 0.1 mmol) were added to the above mixture. The reaction was heated at 110ºC for 8 h and added with 0.4 mL CDCl_3_, then measured by ^1^H NMR spectroscopy.

**For synthesis of** **6ca, 6da, 6ha, 6ia, 6ka, 6la, 6ra, 7da**

In a glovebox, benzoazole (0.1 mmol) and B(C_6_F_5_)_3_ (2.6 mg, 0.005 mmol) were dissolved in CDCl_3_ (0.6 mL) in a 2-mL NMR tube, then Ph_2_SiH_2_ (73.6 mg, 0.4 mmol) and mesitylene (12.0 mg, 0.1 mmol) were added to the above mixture. The reaction was heatedat 110ºC for 8 h, then measured by ^1^H NMR spectroscopy.

**For synthesis of** **6va**, **6wa**, **6ab**, **7ab**, **8ab**

In a glovebox, benzoazole (0.1 mmol) and B(C_6_F_5_)_3_ (2.6 mg, 0.005 mmol) were dissolved in CDCl_3_ (0.6 mL) in a 2-mL NMR tube, then silane (0.2 mmol) and mesitylene (12.0 mg, 0.1 mmol) were added to the above mixture. The reaction was heated at 110ºC for 8 h, then measured by ^1^H NMR spectroscopy.

**For synthesis of** **6ae**, **6ge**, **6pe**, **7ke**, **8ae**

In a glovebox, benzoazole (0.1 mmol) and B(C_6_F_5_)_3_ (2.6 mg, 0.005 mmol) were dissolved in C_6_D_6_ (0.6 mL) in a 2-mL NMR tube, then 4,4'-biphenyl-disilane (10.7 mg, 0.05 mmol) and mesitylene (12.0 mg, 0.1 mmol) were added to the above mixture. The reaction was heated at 110ºC for 8 h, then measured by ^1^H NMR spectroscopy (When 4,4'-biphenyl-disilane was in use, the product yield was higher by using C_6_D_6_ instead of CDCl_3_).

**Typical isolation procedure for solid product 6aa**-**6ha**, **6ja**, **6ka**, **6ma**-**6pa**, **6ae**, **6pe**, **6ge**, **7ba**-**7ka**, **7ke**, **8aa**, **8ab**, **8ae**

In a glovebox, the reaction mixture was concentrated under vacuum, then slowly added with dry hexane (1 mL × 3), removed the up-layer of hexane, dried in vacuo to afford the final product.

**Typical isolation procedure for oil product 6ia**, **6qa**, **6ra**, **6sa**, **6ta**, **6ab-6ad**, **7aa**, **7ab-7ad**, **8ab-8ad**

After the reaction mixture was added into micro vacuum distillation device, it was moved to the outside of glovebox, concentrated under vacuum at RT and removed mesitylene or other by-products quickly at 70°C, 500 mTorr. The device was transferred back to the glovebox and added with 0.5 mL hexane, then put in a refrigerator at -35°C for 24 h. The up-layer of hexane was collected and dried in vacuo to afford the final product.

1. **Gram-scale reaction**

In a glovebox, **1a** (1.35 g, 10 mmol) and B(C_6_F_5_)_3_ (51.2 mg, 0.1 mmol) were dissolved in CHCl_3_ (2 mL) in a 20 mL pressure tube, then added with silane (1.84 g, 10 mmol). After heating the reaction at 110ºC for 24 h, a small aliquot was taken for ^1^H NMR measurement. The reaction mixture was concentrated under vacuum and slowly added with dry hexane (5 mL× 3) and then removed the up-layer of hexane, dried in vacuo to afford the final product (97% isolation yield).

# NMR data for product

3-methyl-2,2-diphenyl-2,3-dihydrobenzo[d][1,3,2]thiazasilole (**6aa**) White solid (97%) ^1^H NMR (500 MHz, CDCl_3_) δ 7.81 – 7.83 (m, 4H, HAr), 7.51 – 7.59 (m, 6H, HAr), 7.43 (dd, *J_1_* = 7.5 Hz, *J_2_* = 1.0 Hz, 1H, HAr), 7.18 (td, *J_1_* = 7.5 Hz, *J_2_* = 1.0 Hz, 1H, HAr), 6.81 (td, *J_1_* = 7.5 Hz, *J_2_* = 0.5 Hz, 1H, HAr), 6.72 (d, *J* = 9.0 Hz, 1H, HAr), 3.04 (s, 3H, NCH_3_). ^13^C NMR (126 MHz, CDCl_3_) δ 148.2, 135.5(4C), 132.4(2C), 131.1(2C), 128.3(4C), 127.4, 125.5, 124.5, 118.1, 109.4, 30.8. ^29^Si NMR (99 MHz, CDCl_3_) δ 9.81

6-methoxy-3-methyl-2,2-diphenyl-2,3-dihydrobenzo[d][1,3,2]thiazasilole (**6ba**) Yellow solid (65%) ^1^H NMR (500 MHz, CDCl_3_) δ 7.79 – 7.81 (m, 4H, HAr), 7.56 – 7.60 (m, 2H, HAr), 7.50 – 7.53 (m, 4H, HAr), 7.10 (d, *J* = 2.5 Hz, 1H, HAr), 6.75 (dd, *J_1_* = 9.0 Hz, *J_2_* = 3.0 Hz, 1H, HAr), 6.60 (d, *J* = 9.0 Hz, 1H, HAr), 3.83 (s, 3H, OCH_3_), 3.00 (s, 3H, NCH_3_). ^13^C NMR (126 MHz, CDCl_3_) δ 152.3, 142.4, 135.5(4C), 132.5(2C), 131.1(2C), 128.3(4C), 125.4, 114.3, 110.8, 109.3, 56.1, 31.0. ^29^Si NMR (99 MHz, CDCl_3_) δ 9.83.

6-bromo-3-methyl-2,2-diphenyl-2,3-dihydrobenzo[d][1,3,2]thiazasilole (**6ca**) White solid (85%) ^1^H NMR (500 MHz, CDCl_3_) δ 7.76 – 7.78 (m, 4H, HAr), 7.56 – 7.59 (m, 2H, HAr), 7.50 – 7.53 (m, 5H, HAr), 7.23 (dd, *J_1_* = 8.5 Hz, *J_2_* = 2.0 Hz, 1H, HAr), 6.53 (d, *J* = 8.5 Hz, 1H, HAr), 2.98 (s, 3H, NCH_3_). ^13^C NMR (126 MHz, CDCl_3_) δ 147.4, 135.4(4C), 131.8(2C), 131.3(2C), 129.5, 128.4(4C), 128.1, 126.8, 110.5, 109.5, 30.9. ^29^Si NMR (99 MHz, CDCl_3_) δ 10.65.

5-bromo-3-methyl-2,2-diphenyl-2,3-dihydrobenzo[d][1,3,2]thiazasilole (**6da**) Yellow solid (92%) ^1^H NMR (500 MHz, CDCl_3_) δ 7.75 – 7.76 (m, 4H, HAr), 7.55 – 7.58 (m, 2H, HAr), 7.49 – 7.52 (m, 4H, HAr), 7.22 (d, *J* = 8.0 Hz, 1H, HAr), 6.88 (dd, *J_1_* = 8.0 Hz, *J_2_* = 1.5 Hz, 1H, HAr), 6.79 (d, *J* = 1.5 Hz, 1H, HAr), 2.97 (s, 3H, NCH_3_), ^13^C NMR (126 MHz, CDCl_3_) δ 149.5, 135.4(4C), 131.7(2C), 131.3(2C), 128.4(4C), 128.2, 123.7, 120.7, 118.9, 112.6, 30.9. ^29^Si NMR (99 MHz, CDCl_3_) δ 11.37.

5-chloro-3-methyl-2,2-diphenyl-2,3-dihydrobenzo[d][1,3,2]thiazasilole (**6ea**) White solid (95%) ^1^H NMR (500 MHz, CDCl_3_) δ 7.78 – 7.79 (m, 4H, HAr), 7.51 – 7.60 (m, 6H, HAr), 7.29 (d, *J* = 8.0 Hz, 1H, HAr), 6.76 (dd, *J_1_* = 8.0 Hz, *J_2_* = 1.5 Hz, 1H, HAr), 6.68 (d, *J* = 2.5 Hz, 1H, HAr), 2.99 (s, 3H, NCH_3_). ^13^C NMR (126 MHz, CDCl_3_) δ 149.3, 135.4(4C), 131.8(2C), 131.3(2C), 128.4(4C), 127.8, 123.0, 117.8(2C), 109.9, 30.9. ^29^Si NMR (99 MHz, CDCl_3_) δ 11.66.

3-methyl-2,2-diphenyl-5-(thiophen-2-yl)-2,3-dihydrobenzo[d][1,3,2]thiazasilole (**6fa**) White solid (90%) ^1^H NMR (500 MHz, CDCl_3_) δ 7.78 – 7.79 (m, 4H, HAr), 7.54 – 7.57 (m, 2H, HAr), 7.49 – 7.52 (m, 4H, HAr), 7.38 (d, *J* = 8.0 Hz, 1H, HAr), 7.32 (d, *J* = 3.5 Hz, 1H, HAr), 7.27 (d, *J* = 5.0 Hz, 1H, HAr), 7.10 (dd, *J_1_* = 8.0 Hz, *J_2_* = 1.5 Hz, 1H, HAr),7.05 (dd, *J_1_* = 8.0 Hz, *J_2_* = 1.5 Hz, 1H, HAr), 6.90 (d, *J* = 1.5 Hz, 1H, HAr), 3.06 (s, 3H, NCH_3_). ^13^C NMR (126 MHz, CDCl_3_) δ 148.5, 145.2, 135.5(4C), 132.1(2C), 131.2(2C), 128.3(4C), 128.1, 127.9, 127.5, 124.2, 124.1, 122.6, 116.2, 107.1, 30.8. ^29^Si NMR (99 MHz, CDCl_3_) δ 10.53.

(5-benzo[b]thiophen-2-yl)-3-methyl-2,2-diphenyl-2,3-dihydrobenzo[d][1,3,2]thiazasilole (**6ga**) White solid (88%) ^1^H NMR (500 MHz, CDCl_3_) δ 7.86 (d, *J* = 8.0 Hz, 1H, HAr), 7.81 – 7.82 (m, 5H, HAr), 7.56 – 7.59 (m, 3H, HAr), 7.51 – 7.54 (m, 4H, HAr), 7.44 (d, *J* = 8.0 Hz, 1H, HAr), 7.38 (td, *J_1_* = 8.0 Hz, *J_2_* = 1.0 Hz, 1H, HAr), 7.33 (td, *J_1_* = 8.0 Hz, *J_2_* = 0.5 Hz, 1H, HAr), 7.17 (dd, *J_1_* = 8.0 Hz, *J_2_* = 1.5 Hz, 1H, HAr), 7.02 (d, *J* = 1.5 Hz, 1H, HAr), 3.10 (s, 3H, NCH_3_). ^13^C NMR (126 MHz, CDCl_3_) δ 148.6, 145.1, 140.8, 139.3, 135.5(4C), 132.0(2C), 131.9, 131.3(2C), 128.4(4C), 127.6, 125.4, 124.5, 124.1, 123.4, 122.2, 118.8, 116.7, 107.4, 30.9. ^29^Si NMR (99 MHz, CDCl_3_) δ 10.67.

1. ([1,1'-biphenyl]-4-yl)-3-methyl-2,2-diphenyl-2,3-dihydrobenzo[d][1,3,2]thiazasilole (**6ha**) White solid (75%) ^1^H NMR (500 MHz, CDCl_3_) δ 7.81 – 7.83 (m, 4H, HAr), 7.70 – 7.76 (m, 5H, HAr), 7.50 – 7.59 (m, 10H, HAr), 7.41 (d, *J* = 7.5 Hz, 1H, HAr), 7.08 (dd, *J_1_* = 8.0 Hz, *J_2_* = 2.0 Hz, 1H, HAr), 6.96 (d, *J* = 1.5 Hz, 1H, HAr), 3.10 (s, 3H, NCH_3_). ^13^C NMR (126 MHz, CDCl_3_) δ 148.6, 140.8, 140.7, 139.8, 138.5, 135.5(4C), 134.1(2C), 132.2(2C), 131.8, 131.2(2C), 128.8(2C), 128.3(4C), 127.6, 127.4(2C), 127.3, 127.1(2C), 117.1, 108.1, 30.9. ^29^Si NMR (99 MHz, CDCl_3_) δ 10.49.

3-methyl-6-(naphthalen-2-yl)-2,2-diphenyl-2,3-dihydrobenzo[d][1,3,2]thiazasilole (**6ia**) Black oil (60%) ^1^H NMR (500 MHz, CDCl_3_) δ 8.00 (d, *J* = 8.5 Hz, 1H, HAr), 7.92 (d, *J* = 8.0 Hz, 1H, HAr), 7.84 (d, *J* = 8.5 Hz, 1H, HAr), 7.63 – 7.65 (m, 3H, HAr), 7.61 (d, *J* = 2.0 Hz, 1H, HAr), 7.40 – 7.55 (m, 9H, HAr), 7.31 – 7.40 (m, 3H, HAr), 6.77 (d, *J* = 8.5 Hz, 1H, HAr), 3.03 (s, 3H, NCH_3_). ^13^C NMR (126 MHz, CDCl_3_) δ 148.7, 139.7, 136.5, 134.4(4C), 134.0, 131.8, 131.3(2C), 130.4(2C), 128.3, 127.8(4C), 127.6, 127.5, 127.1, 126.8, 126.1, 125.9, 125.7, 125.4, 111.0, 109.5, 30.8. ^29^Si NMR (99 MHz, CDCl_3_) δ 10.20.

3-methyl-2,2-diphenyl-6-(4,4,5,5-tetramethyl-1,3,2-dioxaborolan-2-yl)-2,3-dihydrobenzo[d][1,3,2]thiazasilole (**6ja**) White solid (80%) ^1^H NMR (500 MHz, CDCl_3_) δ 7.77 – 7.78 (m, 4H, HAr), 7.54 – 7.56 (m, 2H, HAr), 7.48 – 7.51 (m, 4H, HAr), 7.44 (d, *J* = 7.5 Hz, 1H, HAr), 7.29 (d, *J* = 7.0 Hz, 1H, HAr), 7.14 (s, 1H, HAr), 3.06 (s, 3H, NCH_3_). 1.41 (s, 12H, BpinCH_3_). ^13^C NMR (126 MHz, CDCl_3_) δ 147.6, 135.5(4C), 132.3(2C), 131.1(2C), 129.0, 128.3(4C), 126.9, 125.1, 114.7, 83.6, 30.9(2C), 29.8, 24.9(4C). ^29^Si NMR (99 MHz, CDCl_3_) δ 10.33.

6-(3,5-bis(trifluoromethyl)phenyl)-3-methyl-2,2-diphenyl-2,3-dihydrobenzo[d][1,3,2]thiazasilole (**6ka**) Gray solid (95%) ^1^H NMR (500 MHz, CDCl_3_) δ 8.07 (s, 2H, HAr), 7.87 (s, 1H, HAr), 7.84 – 7.85 (m, 4H, HAr), 7.68 – 7.77 (m, 1H, HAr), 7.59 – 7.62 (m, 2H, HAr), 7.54 – 7.57 (m, 4H, HAr), 7.44 (dd, *J_1_* = 8.0 Hz, *J_2_* = 2.0 Hz, 1H, HAr), 6.82 (d, *J* = 8.5 Hz, 1H, HAr), 3.09 (s, 3H, NCH_3_). ^13^C NMR (126 MHz, CDCl_3_) δ 149.1, 143.1, 135.5(4C), 134.6, 131.8(2C), 131.6(q *J* _C, F_ = 32.8 Hz, 2C), 131.4(2C), 128.5(4C), 128.2, 126.3(2C), 125.9, 124.7, 119.8, 109.8, 30.9(3C). ^19^F NMR (471 MHz, CDCl_3_) δ -62.8. ^29^Si NMR (99 MHz, CDCl_3_) δ 10.66.

1. ethyl-2,2-diphenyl-2,3-dihydrobenzo[d][1,3,2]thiazasilole (**6ma**) White solid (95%) ^1^H NMR (500 MHz, CDCl_3_) δ 7.80 – 7.82 (m, 4H, HAr), 7.47 – 7.56 (m, 6H, HAr), 7.39 (d, *J* = 7.5 Hz, 1H, HAr), 7.11 (td, *J_1_* = 8.0 Hz, *J_2_* = 0.5 Hz, 1H, HAr), 6.73 – 6.78 (m, 2H, HAr), 3.51 – 3.55 (q, 2H, CH_2_), 1.02 (t, *J* = 6.0 Hz, 3H, CH_3_). ^13^C NMR (126 MHz, CDCl_3_) δ 146.8, 135.6(4C), 132.6(2C), 131.0(2C), 128.2(4C), 127.7, 125.3, 124.8, 117.9, 109.9, 38.5, 13.6. ^29^Si NMR (99 MHz, CDCl_3_) δ 8.67.

5-chloro-3-ethyl-2,2-diphenyl-2,3-dihydrobenzo[d][1,3,2]thiazasilole (**6na**) White solid (92%) ^1^H NMR (500 MHz, CDCl_3_) δ 7.78 – 7.80 (m, 4H, HAr), 7.48 – 7.57 (m, 6H, HAr), 7.26 (d, *J* = 9.0 Hz, 1H, HAr), 6.72 (dd, *J_1_* = 9.0 Hz, *J_2_* = 2.0 Hz, 1H, HAr), 6.68 (d, *J* = 2.0 Hz, 1H, HAr), 3.46 – 3.51 (q, 2H, CH_2_), 1.01 (t, *J* = 7.0 Hz, 3H, CH_3_). ^13^C NMR (126 MHz, CDCl_3_) δ 148.0, 135.5(4C), 132.0(2C), 131.2(2C), 131.1, 128.3(4C), 128.1, 123.3, 117.6, 110.2, 38.6, 13.4. ^29^Si NMR (99 MHz, CDCl_3_) δ 10.52.

3-ethyl-5,6-dimethyl-2,2-diphenyl-2,3-dihydrobenzo[d][1,3,2] thiazasilole (**6oa**) White solid (95%) ^1^H NMR (500 MHz, CDCl_3_) 7.79 – 7.81 (m, 4H, HAr), 7.46 – 7.54 (m, 6H, HAr), 7.18 (s, 1H, HAr), 6.55 (s, 1H, HAr), 3.48 – 3.52 (q, 2H, CH_2_), 2.29 (s, 3H, CH_3_), 2.23 (s, 3H, CH_3_), 1.02 (t, *J* = 7.0 Hz, 3H, CH_3_). ^13^C NMR (126 MHz, CDCl_3_) δ 144.7, 135.6(4C), 133.3, 132.8(2C), 130.9(2C), 128.7, 128.2(4C), 125.8, 121.1, 111.5, 38.5, 20.1, 18.8, 13.8. ^29^Si NMR (99 MHz, CDCl_3_) δ 8.97.

1-ethyl-2,2-diphenyl-1,2-dihydronaphtho[1,2-d][1,3,2]thiazasilole (**6pa**) White solid (86%) ^1^H NMR (500 MHz, CDCl_3_) δ 8.21 (d, *J* = 9.0 Hz, 1H, HAr), 7.83 – 7.89 (m, 5H, HAr), 7.41 – 7.57 (m, 10H, HAr), 3.70 – 3.75 (q, 2H, CH_2_), 1.11 (t, *J* = 7.0 Hz, 3H, CH_3_). ^13^C NMR (126 MHz, CDCl_3_) δ 142.6, 135.7(4C), 133.5, 132.2(2C), 131.1(2C), 129.1, 128.2(4C), 126.7, 126.5(2C), 125.0, 124.4, 122.7, 121.8, 43.9, 16.6. ^29^Si NMR (99 MHz, CDCl_3_) δ 13.36.

2,2-diphenyl-3-propyl-2,3-dihydrobenzo[d][1,3,2]thiazasilole (**6qa**) Colorless oil (79%) ^1^H NMR (500 MHz, CDCl_3_) 7.82 – 7.83 (m, 4H, HAr), 7.49 – 7.57 (m, 6H, HAr), 7.41(d, *J* = 7.5 Hz, 1H, HAr), 7.13 (td, *J_1_* = 8.0 Hz, *J_2_* = 1.0 Hz, 1H, HAr), 6.76 (t, *J* = 7.5 Hz, 1H, HAr), 6.72 (d, *J* = 8.0 Hz, 1H, HAr), 3.39 – 3.42 (m, 2H, CH_2_), 1.45 – 1.52 (m, 2H, CH_2_), 0.76 (t, *J* = 7.5 Hz, 3H, CH_3_). ^13^C NMR (126 MHz, CDCl_3_) δ 147.1, 135.6(4C), 132.6(2C), 131.0(2C), 128.2(4C), 127.7, 125.3, 124.7, 117.9, 110.0, 46.2, 21.3, 11.4. ^29^Si NMR (99 MHz, CDCl_3_) δ 9.02.

1. isobutyl-2,2-diphenyl-2,3-dihydrobenzo[d][1,3,2]thiazasilole (**6ra**) Colorless oil (30%) ^1^H NMR (500 MHz, CDCl_3_) 7.80 – 7.82 (m, 4H, HAr), 7.47 – 7.56 (m, 6H, HAr), 7.40 (d, *J* = 7.5 Hz, 1H, HAr), 7.12 (d, *J* = 7.5 Hz, 1H, HAr), 6.75 – 6.79 (m, 2H, HAr), 3.29 (d, *J* = 7.0 Hz, 2H, CH_2_), 2.02 – 2.10 (m, 1H, CH), 0.62 (d, *J* = 6.5 Hz, 6H, CH_3_), ^13^C NMR (126 MHz, CDCl_3_) δ 147.6, 135.8(4C), 132.6(2C), 131.1(2C), 128.2(4C), 127.9, 125.2, 124.4, 118.0, 110.6, 52.4, 26.4, 20.4(2C). ^29^Si NMR (99 MHz, CDCl_3_) δ 10.54.

3-(cyclopropylmethyl)-2,2-diphenyl-2,3-dihydrobenzo[d][1,3,2]thiazasilole (**6sa**) Colorless oil (60%) ^1^H NMR (500 MHz, CDCl_3_) 7.83 – 7.85 (m, 4H, HAr), 7.48 – 7.57 (m, 6H, HAr), 7.40 (dd, *J_1_* = 7.5 Hz, *J_2_* = 1.0 Hz, 1H, HAr), 7.15 (td, *J_1_* = 8.0 Hz, *J_2_* = 1.5 Hz, 1H, HAr), 6.89 (d, *J* = 8.0 Hz, 1H, HAr), 6.78 (t, *J* = 7.5 Hz, 1H, HAr), 3.31 (d, *J* = 7.0 Hz, 2H, CH_2_), 1.06 – 1.14 (m, 1H, CH_2_), 0.27 – 0.31 (m, 2H, CH_2_), -0.19 – -0.15 (m, 2H, CH_2_). ^13^C NMR (126 MHz, CDCl_3_) δ 147.5, 135.8(4C), 132.8(2C), 131.0(2C), 128.2(4C), 127.7, 125.3, 124.6, 118.1, 110.4, 49.5, 10.1, 4.9(2C). ^29^Si NMR (99 MHz, CDCl_3_) δ 9.31.

3-(cyclohexylmethyl)-2,2-diphenyl-2,3-dihydrobenzo[d][1,3,2]thiazasilole (**6ta**) Colorless oil (50%) ^1^H NMR (500 MHz, CDCl_3_) 7.80 – 7.81 (m, 4H, HAr), 7.53 – 7.56 (m, 2H, HAr), 7.47 – 7.50 (m, 4H, HAr), 7.39 (dd, *J_1_* = 6.0 Hz, *J_2_* = 1.5 Hz, 1H, HAr), 7.12 (td, *J_1_* = 9.0 Hz, *J_2_* = 1.5 Hz, 1H, HAr), 6.73 – 6.78 (m, 2H, HAr), 3.29 (d, *J* = 7.0 Hz, 2H, CH_2_), 1.61 – 1.69 (m, 1H, CH), 1.46 – 1.54 (m, 5H, CH_2_), 0.94 – 1.05 (m, 3H, CH_2_), 0.48 – 0.55 (m, 2H, CH_2_). ^13^C NMR (126 MHz, CDCl_3_) δ 147.7, 135.8(4C), 132.6(2C), 131.1(2C), 128.2(4C), 127.8, 125.2, 124.4, 117.9, 110.6, 51.2, 36.0, 31.2(2C), 26.3, 25.9(2C). ^29^Si NMR (99 MHz, CDCl_3_) δ 10.32.

2,2-diethyl-3-methyl-2,3-dihydrobenzo[d][1,3,2]thiazasilole (**6ab**) Colorless oil (85%) ^1^H NMR (500 MHz, CDCl_3_) δ 7.30 (d, *J* = 7.5 Hz, 1H, HAr), 7.06 (t, *J* = 7.5 Hz, 1H, HAr), 6.67 (t, *J* = 8.0 Hz, 1H, HAr), 6.56 (d, *J* = 8.0 Hz, 1H, HAr), 2.93 (s, 3H, NCH_3_), 0.94 – 1.08 (m, 10H, SiCH_2_CH_3_), ^13^C NMR (126 MHz, CDCl_3_) δ 149.2, 127.3, 125.0, 124.9, 117.4, 108.6, 30.1, 8.9(2C), 6.5(2C). ^29^Si NMR (99 MHz, CDCl_3_) δ 35.74.

2,3-dimethyl-2-phenyl-2,3-dihydrobenzo[d][1,3,2]thiazasilole (**6ac**) Colorless oil (90%) ^1^H NMR (500 MHz, CDCl_3_) δ 7.70 – 7.72 (m, 2H, HAr), 7.45 – 7.53 (m, 3H, HAr), 7.36 (dd, *J_1_* = 7.5 Hz, *J_2_* = 1.0 Hz, 1H, HAr), 7.12 (td, *J_1_* = 8.0 Hz, *J_2_* = 1.0 Hz, 1H, HAr), 6.75 (td, *J_1_* = 7.5 Hz, *J_2_* = 0.5 Hz, 1H, HAr), 6.63 (d, *J* = 8.0 Hz, 1H, HAr), 2.89 (s, 3H, NCH_3_), 0.96 (s, 3H, SiCH_3_). ^13^C NMR (126 MHz, CDCl_3_) δ 148.3, 134.9, 134.4(2C), 131.0, 128.2(2C), 127.3, 125.3, 124.8, 117.8, 109.1, 30.0, -0.04. ^29^Si NMR (99 MHz, CDCl_3_) δ 19.24.

3-methyl-2-phenyl-2,3-dihydrobenzo[d][1,3,2]thiazasilole (**6ad**) Colorless oil (74%) ^1^H NMR (500 MHz, CDCl_3_) δ 7.78 – 7.79 (m, 2H, HAr), 7.57 – 7.60 (m, 1H, HAr), 7.50 – 7.53 (m, 2H, HAr), 7.38 (dd, *J_1_* = 6.0 Hz, *J_2_* = 1.0 Hz, 1H, HAr), 7.14 (td, *J_1_* = 7.5 Hz, *J_2_* = 1.0 Hz, 1H, HAr), 6.77 (td, *J_1_* = 7.5 Hz, *J_2_* = 0.5 Hz, 1H, HAr), 6.66 (d, *J* = 8.0 Hz, 1H, HAr), 6.22 (s, 1H, SiH), 2.91 (s, 3H, NCH_3_). ^13^C NMR (126 MHz, CDCl_3_) δ 148.0, 134.8(2C), 133.0, 131.9, 128.4(2C), 127.3, 125.5, 125.0, 118.0, 109.5, 30.7. ^29^Si NMR (99 MHz, CDCl_3_) δ 5.24.

4,4'-bis(3-methyl-2,3-dihydrobenzo[d][1,3,2]thiazasilol-2-yl)-1,1'-biphenyl (**6ae**) White solid (82%) ^1^H NMR (500 MHz, C_6_D_6_) δ 7.54 – 7.55 (m, 4H, HAr), 7.38 – 7.40 (m, 6H, HAr), 7.02 (td, *J_1_* = 8.0 Hz, *J_2_* = 1.5 Hz, 2H, HAr), 6.67 (td, *J_1_* = 8.0 Hz, *J_2_* = 1.0 Hz, 2H, HAr), 6.39 (d, *J* = 8.0 Hz, 2H, HAr), 6.13 (s, 2H, SiH), 2.39 (s, 6H, NCH_3_). ^13^C NMR (126 MHz, C_6_D_6_) δ 147.9(2C), 143.8(2C), 135.3(4C), 132.5(2C), 127.4(2C), 127.1(4C), 125.6(2C), 125.1(2C), 118.4(2C), 109.7(2C), 30.0(2C). ^29^Si NMR (99 MHz, C_6_D_6_) δ 4.66.

4,4'-bis(5-(benzo[b]thiophen-2-yl)-3-methyl-2,3-dihydrobenzo[d][1,3,2]thiazasilol-2-yl)-1,1'-biphenyl (**6ge**) White solid (65%) ^1^H NMR (500 MHz, C_6_D_6_) δ 7.57 – 7.60 (m, 8H, HAr), 7.42 – 7.44 (m, 4H, HAr), 7.35 (s, 2H, HAr), 7.33 (d, *J* = 8.0 Hz, 2H, HAr), 7.18 (t, *J* = 8.0 Hz, 2H, HAr), 7.10 (dd, *J_1_* = 7.5 Hz, *J_2_* = 1.5 Hz, 2H, HAr), 7.06 (t, *J* = 8.0 Hz, 2H, HAr), 6.89 (d, *J* = 1.5 Hz, 2H, HAr), 6.14 (s, 2H, SiH), 2.40 (s, 6H, NCH_3_). ^13^C NMR (126 MHz, C_6_D_6_) δ 148.5(2C), 145.0(2C), 143.9(2C), 141.0(2C), 139.5(2C), 135.3(4C), 132.2(2C), 132.2(2C), 127.6(2C), 127.2(4C), 125.9(2C), 124.5(2C), 124.2(2C), 123.4(2C), 122.2(2C), 119.2(2C), 117.0(2C), 107.9(2C), 29.9(2C). ^29^Si NMR (99 MHz, C_6_D_6_) δ 5.61.

4,4'-bis(1-ethyl-1,2-dihydronaphtho[1,2-d][1,3,2]thiazasilol-2-yl)-1,1'-biphenyl (**6pe**) White solid (88%) ^1^H NMR (500 MHz, C_6_D_6_) δ 8.00 (d, *J* = 8.5 Hz, 2H, HAr), 7.60 – 7.66 (m, 6H, HAr), 7.50 (d, *J* = 8.5 Hz, 2H, HAr), 7.31 (d, *J* = 8.5 Hz, 2H, HAr), 7.15 – 7.23 (m, 8H, HAr), 6.15 (s, 2H, SiH), 3.47 – 3.55 (m, 4H, CH_2_), 0.97 (t, *J* = 7.5 Hz, 6H, CH_3_). ^13^C NMR (126 MHz, C_6_D_6_) δ 143.4(2C), 142.3(2C), 134.8(4C), 133.7(2C), 133.3(2C), 129.2(2C), 127.1(4C), 126.6(2C), 126.2(2C), 124.9(2C), 124.5(2C), 124.4(2C), 122.5(2C), 122.0(2C), 44.9(2C), 16.0(2C). ^29^Si NMR (99 MHz, C_6_D_6_) δ 7.99.

3-methyl-2,2-diphenyl-2,3-dihydrobenzo[d][1,3,2]oxazasilole (**7aa**) colorless oil (69%) ^1^H NMR (500 MHz, CDCl_3_) δ 7.74 (d, *J* = 10.0 Hz, 4H, HAr), 7.57 (t, *J* = 5.0 Hz, 2H, HAr), 7.48 (d, *J* = 10.0 Hz, 4H, HAr), 7.01 (d, *J* = 5.0 Hz, 1H, HAr), 6.96 (t, *J* = 10.0 Hz, 1H, HAr), 6.75 (t, *J* = 5.0 Hz, 1H, HAr), 6.71 (d, *J* = 10.0 Hz, 1H, HAr), 3.04 (s, 3H, CH_3_), ^13^C NMR (126 MHz, CDCl_3_) δ 149.2, 141.9, 135.2(4C), 131.6(2C), 130.6(2C), 128.3(4C), 121.2, 117.3, 112.4, 107.5, 28.7. ^29^Si NMR (99 MHz, CDCl_3_) δ -4.36.

3,5-dimethyl-2,2-diphenyl-2,3-dihydrobenzo[d][1,3,2]oxazasilole (**7ba**) White solid (90%) ^1^H NMR (500 MHz, CDCl_3_) δ 7.73 – 7.75 (m, 4H, HAr), 7.55 – 7.59 (m, 2H, HAr), 7.47 – 7.50 (m, 4H, HAr), 6.88 (d, *J* = 8.0 Hz, 1H, HAr), 6.55 – 6.56 (m, 2H, HAr), 3.02 (s, 3H, CH_3_), 2.39 (s, 3H, CH_3_). ^13^C NMR (126 MHz, CDCl_3_) δ 147.1, 141.7, 135.2(4C), 131.5(2C), 130.7(2C), 128.2(4C), 128.0, 117.3, 111.9, 108.6, 28.7, 21.5. ^29^Si NMR (99 MHz, CDCl_3_) δ -4.02.

3,6-dimethyl-2,2-diphenyl-2,3-dihydrobenzo[d][1,3,2]oxazasilole (**7ca**) White solid (89%) ^1^H NMR (500 MHz, CDCl_3_) δ 7.74 – 7.77 (m, 4H, HAr), 7.46 – 7.55 (m, 6H, HAr), 7.04 (d, *J* = 2.5 Hz, 1H, HAr), 6.70 (dd, *J_1_* = 9.0 Hz, *J_2_* = 2.5 Hz, 1H, HAr), 6.56 (d, *J* = 9.0 Hz, 1H, HAr), 3.80 (s, 3H, CH_3_), 2.97 (s, 3H, CH_3_). ^13^C NMR (126 MHz, CDCl_3_) δ 152.3, 142.4, 135.4(4C), 132.4(2C), 131.0(2C), 128.2(4C), 125.4, 114.2, 110.7, 109.2, 56.1, 31.0. ^29^Si NMR (99 MHz, CDCl_3_) δ -4.21.

5-chloro-3-methyl-2,2-diphenyl-2,3-dihydrobenzo[d][1,3,2]oxazasilole (**7da**) White solid (74%) ^1^H NMR (500 MHz, CDCl_3_) δ 7.73 – 7.75 (m, 4H, HAr), 7.59 – 7.62 (m, 2H, HAr), 7.50 – 7.53 (m, 4H, HAr), 6.89 (d, *J* = 5.0 Hz, 1H, HAr), 6.68 – 6.71 (m, 2H, HAr), 3.01 (s, 3H, CH_3_), ^13^C NMR (126 MHz, CDCl_3_) δ 147.9, 143.2, 135.2(4C), 131.8(2C), 130.0(2C), 128.4(4C), 126.3, 116.7, 112.8, 108.1, 28.8. ^29^Si NMR (99 MHz, CDCl_3_) δ -2.70.

3-methyl-6-nitro-2,2-diphenyl-2,3-dihydrobenzo[d][1,3,2]oxazasilole (**7ea**) Black solid (35%) ^1^H NMR (500 MHz, CDCl_3_) δ 7.99 (dd, *J_1_* = 8.5 Hz, *J_2_* = 2.5 Hz, 1H, HAr), 7.82 (d, *J* = 2.5 Hz, 1H, HAr), 7.70 – 7.72 (m, 4H, HAr), 7.60 – 7.63 (m, 2H, HAr), 7.50 – 7.53 (m, 4H, HAr), 6.65 (d, *J* = 9.0 Hz, 1H, HAr), 3.07 (s, 3H, CH_3_). ^13^C NMR (126 MHz, CDCl_3_) δ 156.5, 148.6, 139.0, 135.1(4C), 134.5(2C), 132.3(2C), 128.6(4C), 119.9, 108.0, 105.9, 29.1. ^29^Si NMR (99 MHz, CDCl_3_) δ -1.23.

3-ethyl-2,2-diphenyl-2,3-dihydrobenzo[d][1,3,2]oxazasilole (**7fa**) White solid (95%) ^1^H NMR (500 MHz, CDCl_3_) δ 7.76 – 7.77 (m, 4H, HAr), 7.55 – 7.59 (m, 2H, HAr), 7.47 – 7.50 (m, 4H, HAr), 6.99 (d, *J* = 7.0 Hz, 1H, HAr), 6.93 (t, *J* = 7.5 Hz, 1H, HAr), 6.72 – 6.75 (m, 2H, HAr), 3.45 – 3.49 (q, 2H, CH_2_), 1.20 (t, *J* = 7.5 Hz, 3H, CH_3_). ^13^C NMR (126 MHz, CDCl_3_) δ 149.2, 141.0, 135.3(4C), 131.4(2C), 131.2(2C), 128.2(4C), 121.1, 117.3, 112.6, 108.0, 37.5, 14.6. ^29^Si NMR (99 MHz, CDCl_3_) δ -4.82.

3-ethyl-5-methyl-2,2-diphenyl-2,3-dihydrobenzo[d][1,3,2]oxazasilole (**7ga**) White solid (55%) ^1^H NMR (500 MHz, CDCl_3_) δ 7.76 – 7.78 (m, 4H, HAr), 7.54 – 7.58 (m, 2H, HAr), 7.47 – 7.50 (m, 4H, HAr), 6.88 (d, *J* = 7.5 Hz, 1H, HAr), 6.57 (s, 1H, HAr), 6.53 (d, *J* = 8.0 Hz, 1H, HAr), 3.44 – 3.48 (q, 2H, CH_2_), 2.38 (s, 3H, CH_3_) 1.20 (t, *J* = 7.0 Hz, 3H, CH_3_). ^13^C NMR (126 MHz, CDCl_3_) δ 147.1, 140.8, 135.3(4C), 134.3, 131.4(2C), 130.5(2C), 128.2(4C), 117.2, 112.1, 109.1, 37.5, 21.5, 14.6. ^29^Si NMR (99 MHz, CDCl_3_) δ -4.54.

5-chloro-3-ethyl-2,2-diphenyl-2,3-dihydrobenzo[d][1,3,2]oxazasilole (**7ha**) White solid (82%) ^1^H NMR (500 MHz, CDCl_3_) δ 7.74 – 7.78 (m, 4H, HAr), 7.58 – 7.61 (m, 2H, HAr), 7.50– 7.53 (m, 4H, HAr), 6.86 – 6.91 (m, 1H, HAr), 6.68 – 6.72 (m, 2H, HAr), 3.41 – 3.47 (m, 2H, CH_2_), 1.18 – 1.23 (m, 3H, CH_3_). ^13^C NMR (126 MHz, CDCl_3_) δ 148.0, 142.2, 135.3(4C), 131.7(2C), 130.6(2C), 128.4(4C), 126.1, 116.6, 112.9, 108.5, 37.7, 14.4. ^29^Si NMR (99 MHz, CDCl_3_) δ -3.20.

3-ethyl-2,2,5-triphenyl-2,3-dihydrobenzo[d][1,3,2]oxazasilole (**7ia**) Yellow solid (88%) ^1^H NMR (500 MHz, CDCl_3_) δ 7.80 – 7.82 (m, 4H, HAr), 7.66 – 7.67 (m, 2H, HAr), 7.58 – 7.61 (m, 2H, HAr), 7.47 – 7.53 (m, 6H, HAr), 7.35 (t, *J* = 7.5 Hz, 1H, HAr), 7.07 (d, *J* = 7.5 Hz, 1H, HAr), 6.97 – 7.00 (m, 2H, HAr), 3.53 – 3.57 (q, 2H, CH_2_), 1.25 (t, *J* = 7.0 Hz, 3H, CH_3_). ^13^C NMR (126 MHz, CDCl_3_) δ 149.1, 142.3, 141.3, 135.3(4C), 134.8, 131.5(2C), 131.1(2C), 128.6(2C), 128.3(4C), 127.1(2C), 126.5, 116.3, 112.6, 107.2, 37.6, 14.6. ^29^Si NMR (99 MHz, CDCl_3_) δ -3.80.

6-bromo-3-ethyl-2,2-diphenyl-2,3-dihydrobenzo[d][1,3,2]oxazasilole (**7ja**) White solid (87%) ^1^H NMR (500 MHz, CDCl_3_) δ 7.76 – 7.78 (m, 4H, HAr), 7.58 – 7.61 (m, 2H, HAr), 7.50– 7.53 (m, 4H, HAr), 7.14 (d, *J* = 2.0 Hz, 1H, HAr), 7.06 (dd, *J_1_* = 9.0 Hz, *J_2_* = 2.0 Hz, 1H, HAr), 6.58 (d, *J* = 8.5 Hz, 1H, HAr), 3.43 – 3.47 (q, 2H, CH_2_), 1.20 (t, *J* = 6.0 Hz, 3H, CH_3_). ^13^C NMR (126 MHz, CDCl_3_) δ 150.1, 140.4, 135.3(4C), 131.7(2C), 130.7(2C), 128.4(4C), 123.7, 116.0, 108.9, 108.6, 37.6, 14.5. ^29^Si NMR (99 MHz, CDCl_3_) δ -3.51.

1-ethyl-2,2-diphenyl-1,2-dihydronaphtho[1,2-d][1,3,2]oxazasilole White solid (92%) (**7ka**) ^1^H NMR (500 MHz, CDCl_3_) δ 8.08 (d, *J* = 8.5 Hz, 1H, HAr), 7.78 – 7.81 (m, 5H, HAr), 7.48 – 7.58 (m, 7H, HAr), 7.40 (t, *J* = 7.5 Hz, 1H, HAr), 7.22 (t, *J* = 7.5 Hz, 1H, HAr), 7.19 (d, *J* = 8.5 Hz, 1H, HAr), 3.55 – 3.60 (m, 2H, CH_2_), 1.24 (t, *J* = 7.5 Hz, 3H, CH_3_). ^13^C NMR (126 MHz, CDCl_3_) δ 142.3, 136.1, 135.4(4C), 131.4(2C), 128.3(4C), 127.9(2C), 127.5, 125.2(2C), 123.0, 122.0, 120.5, 119.7, 111.0, 37.6, 15.2. ^29^Si NMR (99 MHz, CDCl_3_) δ -3.29.

2,2-diethyl-3-methyl-2,3-dihydrobenzo[d][1,3,2]oxazasilole (**7ab**) Colorless oil (81%) ^1^H NMR (500 MHz, CDCl_3_) δ 6.85 – 6.90 (m, 2H, HAr), 6.64 (t, *J* = 9.0 Hz, 1H, HAr), 6.58 (d, *J* = 7.5 Hz, 1H, HAr), 2.94 (s, 3H, NCH_3_), 1.02 (t, *J* = 7.5 Hz, 6H, CH_3_), 0.87 – 0.97 (q, 4H, CH_2_). ^13^C NMR (126 MHz, CDCl_3_) δ 149.8, 142.6, 120.7, 116.6, 111.8, 106.9, 28.5, 6.7(2C), 5.7(2C). ^29^Si NMR (99 MHz, CDCl_3_) δ 24.23.

2,3-dimethyl-2-phenyl-2,3-dihydrobenzo[d][1,3,2]oxazasilole (**7ac**) Colorless oil (82%) ^1^H NMR (500 MHz, CDCl_3_) δ 7.63 – 7.64 (m, 2H, HAr), 7.52 – 7.55 (m, 1H, HAr), 7.44 – 7.47 (m, 2H, HAr), 6.92 – 6.96 (m, 2H, HAr), 6.70 (dd, *J_1_* = 7.5 Hz, *J_2_* = 1.0 Hz, 1H, HAr), 6.65 (d, *J* = 7.5 Hz, 1H, HAr), 2.92 (s, 3H, NCH_3_) 0.81 (s, 3H, SiCH_3_). ^13^C NMR (126 MHz, CDCl_3_) δ 149.2, 142.0, 134.1(2C), 133.1, 131.5, 128.3(2C), 121.0, 117.1, 112.2, 107.3, 28.2, -3.2. ^29^Si NMR (99 MHz, CDCl_3_) δ 9.96.

3-methyl-2-phenyl-2,3-dihydrobenzo[d][1,3,2]oxazasilole (**7ad**) Colorless oil (32%) ^1^H NMR (500 MHz, CDCl_3_) δ 7.70 – 7.72 (m, 2H, HAr), 7.58 – 7.61 (m, 1H, HAr), 7.49 – 7.52 (m, 2H, HAr), 6.93 – 6.97 (m, 2H, HAr), 6.72 (td, *J_1_* = 8.0 Hz, *J_2_* = 1.0 Hz, 1H, HAr), 6.68 (d, *J* = 8.0 Hz, 1H, HAr), 5.95 (s, 1H, SiH), 2.92 (s, 3H, NCH_3_). ^13^C NMR (126 MHz, CDCl_3_) δ 149.4, 141.5, 134.5(2C), 132.4, 131.3, 128.4(2C), 121.3, 117.5, 112.4, 107.7, 28.3. ^29^Si NMR (99 MHz, CDCl_3_) δ -4.06.

4,4'-bis(1-ethyl-1,2-dihydronaphtho[1,2-d][1,3,2]oxazasilol-2-yl)-1,1'-biphenyl (**7ke**) White solid (79%) ^1^H NMR (500 MHz, C_6_D_6_) δ 8.36 (d, *J* = 8.5 Hz, 2H, HAr), 7.75 (d, *J* = 8.5 Hz, 2H, HAr), 7.51 (d, *J* = 8.0 Hz, 4H, HAr), 7.47 (d, *J* = 9.0 Hz, 2H, HAr), 7.34 (d, *J* = 8.0 Hz, 4H, HAr), 7.31 (td, *J_1_* = 6.5 Hz, *J_2_* = 1.0 Hz, 2H, HAr), 7.14 (td, *J_1_* = 7.5 Hz, *J_2_* = 1.0 Hz, 2H, HAr), 6.94 (d, *J* = 8.5 Hz, 2H, HAr), 6.15 (s, 2H, SiH), 2.96 – 3.03 (m, 4H, CH_2_), 0.94 (t, *J* = 7.5 Hz, 6H, CH_3_). ^13^C NMR (126 MHz, C_6_D_6_) δ 144.0(2C), 143.1(2C), 135.8(2C), 134.9(4C), 132.3(2C), 128.8(2C), 128.1(2C), 127.1(4C), 125.6(2C), 123.6(2C), 122.4(2C), 121.0(2C), 119.7(2C), 110.9(2C), 37.2(2C), 14.7(2C). ^29^Si NMR (99 MHz, C_6_D_6_) δ -4.13.

3-methyl-2,2-diphenyl-2,3-dihydrobenzo[d][1,3,2]selenazasilole (**8aa**) Light yellow solid (97%) ^1^H NMR (500 MHz, CDCl_3_) δ 7.79 – 7.81 (m, 4H, HAr), 7.49 – 7.56 (m, 7H, HAr), 7.19 (dd, *J_1_* = 7.5 Hz, *J_2_* = 1.0 Hz, 1H, HAr), 6.75 (t, *J* = 7.5 Hz, 1H, HAr), 6.68 (d, *J* = 8.0 Hz, 1H, HAr), 3.00 (s, 3H, NCH_3_). ^13^C NMR (126 MHz, CDCl_3_) δ 149.2, 135.6(4C), 132.6(2C), 131.0(2C), 129.8, 128.3(4C), 126.2, 120.7, 118.4, 110.4, 31.6. ^29^Si NMR (99 MHz, CDCl_3_) δ 10.79.

3-ethyl-2,2-diphenyl-2,3-dihydrobenzo[d][1,3,2]selenazasilole (**8ba**) Yellow solid (80%) ^1^H NMR δ 7.85 – 7.87 (m, 4H, HAr), 7.49 – 7.57 (m, 7H, HAr), 7.19 (t, *J* = 8.0 Hz, 1H, HAr), 6.74 – 6.78 (m, 2H, HAr), 3.54 – 3.58 (q, 2H, CH_2_), 1.00 (t, *J* = 7.0 Hz, 3H, CH_3_). ^13^C NMR (126 MHz, CDCl_3_) δ 147.7, 135.7(4C), 132.8(2C), 131.0(2C), 130.3, 128.3(4C), 126.0, 121.1, 118.2, 111.0, 39.1, 13.4. ^29^Si NMR (99 MHz, CDCl_3_) δ 9.71.

2,2-diethyl-3-methyl-2,3-dihydrobenzo[d][1,3,2]selenazasilole (**8ab**) Colorless oil (79%) ^1^H NMR (500 MHz, CDCl_3_) δ 7.41 (d, *J* = 7.5 Hz, 1H, HAr), 7.11 (t, *J* = 7.5 Hz, 1H, HAr), 6.65 (t, *J* = 7.5 Hz, 1H, HAr), 6.54 (d, *J* = 8.0 Hz, 1H, HAr), 2.93 (s, 3H, NCH_3_), 1.00 – 1.15 (m, 10H, SiCH_2_CH_3_). ^13^C NMR (126 MHz, CDCl_3_) δ 150.2, 129.9, 125.8, 120.4, 117.7, 109.6, 30.8, 9.3(2C), 6.9(2C). ^29^Si NMR (99 MHz, CDCl_3_) δ 36.82.

2,3-dimethyl-2-phenyl-2,3-dihydrobenzo[d][1,3,2]selenazasilole (**8ac**) Colorless oil (86%) ^1^H NMR (500 MHz, CDCl_3_) δ 7.71 (d, *J* = 7.0 Hz, 2H, HAr), 7.44 – 7.52 (m, 4H, HAr), 7.17 (t, *J* = 8.0 Hz, 1H, HAr), 6.72 (t, *J* = 7.0 Hz, 1H, HAr), 6.61 (d, *J* = 8.0 Hz, 1H, HAr), 2.87 (s, 3H, NCH_3_), 1.05 (s, 3H, SiCH_3_). ^13^C NMR (126 MHz, CDCl_3_) δ 149.3, 135.2, 134.5(2C), 130.9, 129.8, 128.2(2C), 126.1, 120.8, 118.1, 110.1, 30.8, 0.7. ^29^Si NMR (99 MHz, CDCl_3_) δ 18.44.

1. methyl-2-phenyl-2,3-dihydrobenzo[d][1,3,2]selenazasilole (**8ad**) Colorless oil (60%) ^1^H NMR (500 MHz, CDCl_3_) δ 7.79 – 7.80 (m, 2H, HAr), 7.54 – 7.58 (m, 1H, HAr), 7.46 – 7.51 (m, 3H, HAr), 7.17 (t, *J* = 8.0 Hz, 1H, HAr), 6.74 (t, *J* = 7.5 Hz, 1H, HAr), 6.63 (d, *J* = 8.0 Hz, 1H, HAr), 6.24 (s, 1H, SiH), 2.90 (s, 3H, NCH_3_). ^13^C NMR (126 MHz, CDCl_3_) δ 149.0, 134.9(2C), 133.0, 131.7, 129.7, 128.5(2C), 126.2, 121.2, 118.3, 110.5, 31.8. ^29^Si NMR (99 MHz, CDCl_3_) δ 3.69.

4,4'-bis(3-methyl-2,3-dihydrobenzo[d][1,3,2]selenazasilol-2-yl)-1,1'-biphenyl (**8ae**) Brown solid (42%) ^1^H NMR (500 MHz, C_6_D_6_) δ 7.57 (d, *J* = 8.5 Hz, 4H, HAr), 7.41 (dd, *J_1_* = 7.5 Hz, *J_2_* = 1.0 Hz, 2H, HAr), 7.39 (d, *J* = 8.0 Hz, 4H, HAr), 7.05 (td, *J_1_* = 7.5 Hz, *J_2_* = 1.0 Hz, 2H, HAr), 6.64 (td, *J_1_* = 7.5 Hz, *J_2_* = 1.0 Hz, 2H, HAr), 6.36 (d, *J* = 7.5 Hz, 2H, HAr), 6.15 (s, 2H, SiH), 2.39 (s, 6H, NCH_3_). ^13^C NMR (126 MHz, C_6_D_6_) δ 148.9(2C), 143.6(2C), 135.4(4C), 132.5(2C), 129.7(2C), 127.2(4C), 126.2(2C), 121.4(2C), 118.6(2C), 110.7(2C), 31.1(2C). ^29^Si NMR (99 MHz, C_6_D_6_) δ 3.18.

1. **Supplementary Table 1. Optimization of silane insertion reaction**

| Entry^[a]^ | Cat. | Temp  (ºC) | Time  (h) |  | Yield (%)^[b]^ |  |
| --- | --- | --- | --- | --- | --- | --- |
|  |  |  |  | **4a** | **5aa** | **6aa** |
| 1 | B(C_6_F_5_)_3_ | RT | 2 | 5 | 0 | 0 |
| 2 | B(C_6_F_5_)_3_ | 50 | 2 | 5 | 0 | 0 |
| 3 | B(C_6_F_5_)_3_ | 80 | 2 | 5 | 26 | 0 |
| 4 | B(C_6_F_5_)_3_ | 80 | 8 | 5 | 83 | 1 |
| 5 | B(C_6_F_5_)_3_ | 110 | 2 | 5 | 87 | 1 |
| 6 | B(C_6_F_5_)_3_ | 110 | 8 | 4 | 0 | 95 |
| 7 | BF_3_·Et_2_O | 110 | 8 | 0 | 0 | 0 |
| 8 | B(OH)_3_ | 110 | 8 | 0 | 0 | 0 |
| 9 | [Et_3_Si^+^][B(C_6_F_5_)_4_^-^] | 110 | 8 | 0 | 21 | 21 |
| 10 | AlCl_3_ | 110 | 8 | 0 | 0 | 0 |
| 11 | Al(OTf)_3_ | 110 | 8 | 0 | 0 | 0 |
| 12 | Sc(OTf)_3_ | 110 | 8 | 0 | 0 | 0 |
| 13 | Ni(PPh_3_)_2_Cl_2_ | 110 | 8 | 0 | 0 | 0 |

^[a]^ The reaction was performed with 0.1 mmol **1a**, 0.1 mmol Ph_2_SiH_2_ and 5 mol% catalyst in CDCl_3_; ^[b]^ Yields were quantified by ^1^H NMR spectroscopy with mesitylene as internal standard.

# 5. Comparison of the atomic efficiency of our strategy with that of traditional method

By taking **6aa** as example, we compared traditional method using chlorosilane/triethylamine with our method and found both methods obtained similar product yield but the atomic efficiency of traditional method is only 54% whereas ours is 100%. If o-thiophenol was utilized as starting material, the atomic efficiency for traditional method is only 35%, which is much lower than that of our strategy (79%). The calculation process and corresponding references are specified as below.^10-12^ It is also noted that in the traditional method, the intermediate material o-methylaminothiophenol is very easily oxidized to disulfanediylbis(N-methylaniline). This undoubtedly increases the difficulty of the traditional reaction process. All these results demonstrated that our method is far superior to traditional one.

**Supplementary** **Fig. 1** Comparison of the atomic efficiency of (a) our strategy (b) traditional method (taking **6aa** as example).

# 6. Mechanism studies

**6.1 In-situ monitoring of the silane insertion reaction**

In a glove box, B(C_6_F_5_)_3_ (0.0025 mmol, 1.3 mg) was added to a solution containing **1a** (0.1 mmol, 13.5 mg), mesitylene (0.1 mmol, 12.0 mg) and Ph_2_SiH_2_ (0.1 mmol, 18.4 mg) in 0.6 mL CDCl_3_ in NMR tube and heated at 110ºC. It was measured by ^1^H NMR and ^19^F NMR spectroscopy at different intervals.

**Supplementary Fig. 2.** Overlay of ^1^H NMR spectra obtained from the monitoring of reaction (500 MHz, CDCl_3_).

**Supplementary Fig. 3.** Overlay of ^19^F NMR spectra obtained from the monitoring of reaction (471 MHz, CDCl_3_).

## 6.2 Reaction of B(C_6_F_5_)_3_ and 1a

In a glove box, B(C_6_F_5_)_3_ (25.6 mg, 0.05 mmol) was added to a solution containing **1a** (6.8 mg, 0.05 mmol) and 0.6 mL CDCl_3_ in NMR tube, which was measured by ^1^H NMR and ^19^F NMR spectroscopy.

**Supplementary Fig. 4.** Overlay of ^1^H NMR spectra obtained for the reaction of B(C_6_F_5_)_3_ and **1a** (500 MHz, CDCl_3_).

**Supplementary Fig. 5.** Overlay of ^19^F NMR spectra of the reaction of B(C_6_F_5_)_3_ and **1a** (471 MHz, CDCl_3_).

## 6.3 Deuterium-labelled NMR reaction

Scheme a: In a glove box, B(C_6_F_5_)_3_ (2.6 mg, 0.005 mmol) was added to a solution containing **1a** (13.5 mg, 0.1 mmol), mesitylene (12.0 mg, 0.1 mmol) and Ph_2_SiD_2_ (18.6 mg, 0.1 mmol), 0.6 mL CDCl_3_ in NMR tube. After heating at 110ºC for 8 h, it was measured by ^1^H NMR. All deuterium signals were observed at the methyl position of the product.

Scheme b: In a glove box, B(C_6_F_5_)_3_ (2.6 mg, 0.005 mmol) was added to a solution containing **1a-D** (13.6 mg, 0.1 mmol), mesitylene (12.0 mg, 0.1 mmol) and Ph_2_SiH_2_ (18.4 mg, 0.1 mmol) in 0.6 mL CDCl_3_ in NMR tube. Reaction at 110ºC for 8 h. It was measured by ^1^H NMR, all deuterium signals were observed at the methyl position of the product.


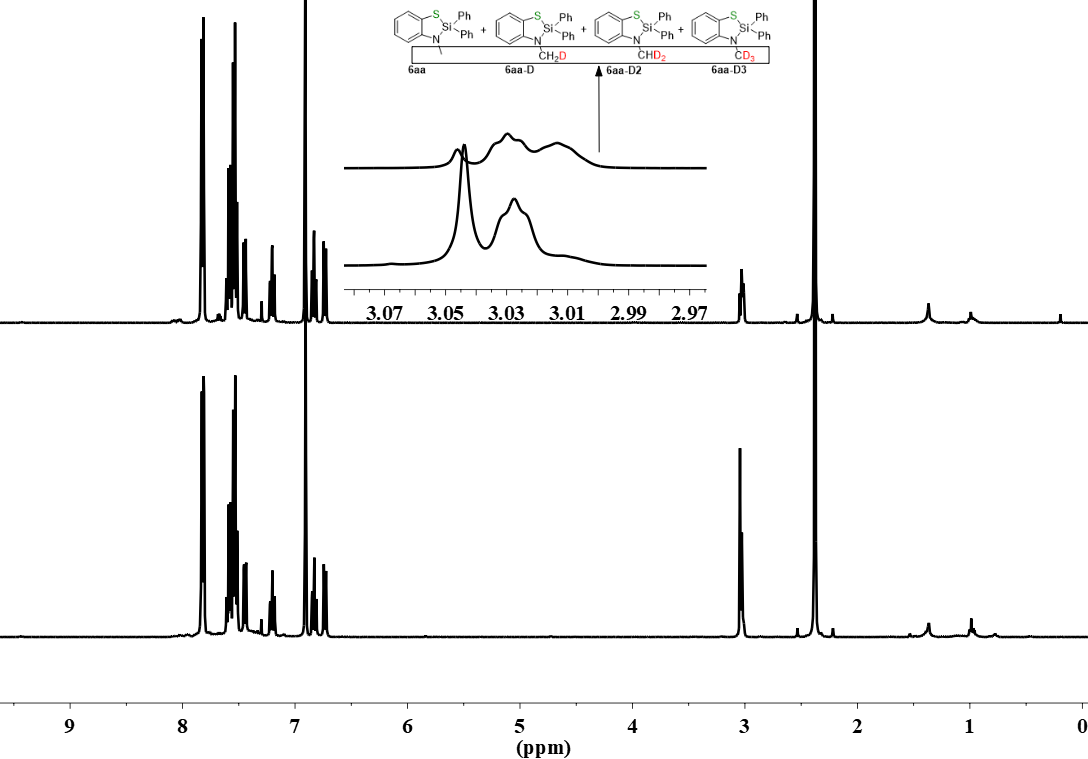


**Supplementary Fig. 6.** Overlay of ^1^H NMR spectra obtained for in-situ reaction as shown in scheme a (top) and scheme b (bottom) (400 MHz, CDCl_3_).

## 6.4 Using tertiary silane as silicon source

In a glove box, B(C_6_F_5_)_3_ (2.6 mg, 0.005 mmol) was added to a solution containing **1a** (13.5 mg, 0.1 mmol), Ph_3_SiH (25.9 mg, 0.1 mmol)/Et_3_SiH (11.6 mg, 0.1 mmol) and 0.6 mL CDCl_3_ in NMR tube. It was heating at 110 °C for 8 h and monitored by ^1^H NMR spectroscopy. No product was obtained by using Ph_3_SiH as silicon source whereas 36% yield of hydrosilylation product was obtained by utilizing Et_3_SiH as silicon source.

**Supplementary Fig. 7.** Overlay of ^1^H NMR spectra of the reaction by employing Ph_3_SiH (top), Et_3_SiH (bottom) (400 MHz, CDCl_3_).

## 6.5 Gibbs free energy profile for the possible hydrosilylation and ring-closing/ring-opening pathway

**Supplementary Fig. 8.** Gibbs free energy profile for the B(C_6_F_5_)_3_ catalyzed silane insertion into benzothiazole through hydrosilylation and ring-closing/ring-opening pathway (in kcal mol^−1^).

The possibility of a ring isomerization mechanism through ring-closing/ring-opening process was also considered. The results (Supplementary Fig. 8.) show that this pathway is thermodynamically less favorable than that discussed in the text.

## 6.6 In-situ NMR reaction heating at 80ºC or 110ºC

In a glove box, B(C_6_F_5_)_3_ (2.6 mg, 0.005 mmol) was added to a solution containing **1a** (13.5 mg, 0.1 mmol), mesitylene (12.0 mg, 0.1 mmol), Ph_2_SiH_2_ (18.4 mg, 0.1 mmol) and 0.6 mL CDCl_3_ in NMR tube. After heating at 80 or 110ºC for 8 h, it was measured by ^1^H NMR spectroscopy, furnishing **4a** in 5%, **5aa** in 83% and **6aa** in 1% yield for heating at 80ºC whereas **4a** in 4%, **6aa** in 95% yield for heating at 110ºC.

**Supplementary Fig. 9.** Overlay of ^1^H NMR spectra of (a) **1a**, (b) reaction heating at 80ºC for 8 h (c) reaction heating at 110ºC for 8 h (400 MHz, CDCl_3_).

**6.7 Reaction of Int5-Me with B(C_6_F_5_)_3_**

In a glove box, **Int5-Me** (33.3 mg, 0.1 mmol) was added to a solution containing 0.6 mL CDCl_3_, and B(C_6_F_5_)_3_ (2.6 mg, 0.005 mmol). After heating at 110ºC for 2 h, it was measured by ^1^H NMR spectroscopy.

**Supplementary Fig. 10.** Overlay of ^1^H NMR spectra of **Int5-Me** (top), reaction of **Int5-Me** and B(C_6_F_5_)_3_ (bottom)(400 MHz, CDCl_3_).

# 7. X-ray single crystal data

Single crystals were quickly covered with a layer of Paratone-N oil (Exxon, dried and degassed at 120ºC/10^-6^ Torr for 24 h) after decanting the mother liquor. A crystal was then mounted on a thin glass fiber and transferred into the cold nitrogen stream of a Bruker APEX-II CCD diffractometer. The structures were solved by direct methods and refined using the Bruker SHELXTL program library by full-matrix least squares on F^2^ for all reflections (SHELXTL, ersion 6.12; Bruker Analytical X-ray Solutions: Madison, WI, 2001). The structure was refined by full-matrix least-squares on *F^2^* for all reflections. All non-hydrogen atoms were refined with anisotropic displacement parameters, whereas hydrogen atoms were included in the structure factor calculations at idealized positions (Sheldrick, G. M. Acta Crystallogr., Sect. A. 1990, 46, 467–473 & 2008, 64, 112–122.).

**Supplementary Fig. 11.** X-ray crystal structure of **6aa**. Hydrogen atoms are omitted for clarity and ellipsoids drawn at 50% probability.

**Supplementary Table 2.** Crystal Data and Structure Refinement for **6aa**

| Compound | **6aa (**2167471**)** |
| --- | --- |
| empirical formula | C_19_H_17_NSSi |
| MW | 319.49 |
| wavelength, Å | 0.71073 |
| crystal system | Monoclinic |
| space group | Cc |
| a, Å | 12.7165(13) |
| b, Å | 20.595(2) |
| c, Å | 6.1703(6) |
| α, deg | 90 |
| b, deg | 100.351(3) |
| g, deg | 90 |
| V, Å^3^ | 1589.7(3) |
| Z | 4 |
| D_calc_, g cm^-3^ | 1.335 |
| µ, mm^-1^ | 0.274 |
| F(000) | 704 |
| crystal size, mm | 0.11 x 0.10 x 0.09 |
| q range, deg | 3.257 to 26.734 |
| limiting indices | 16<=h<=16, -26<=k<=26, -7<=l<=7 |
| reflns collected | 15322 |
| independent reflns | 3166 [R(int) = 0.0185] |
| absorption correction | none |
| data/restraints/para’s | 3166 / 2 / 200 |
| goodness-of-fit on F^2^ | 1.039 |
| final R indices | R1 = 0.0190 |
| [ I >2σ(I)] ^[a]^ | wR2 = 0.0486 |
| R indices (all data) ^[a]^ | R1 = 0.0193, wR2 = 0.0489 |
| peak_max_/hole_min_ (e Å^-3^) | 0.222/-0.137 |

^[a]^ R1 = Σ||F_o_|–|F_c_||/Σ|F_o_|; wR2 = {Σ [w (F_o_^2^–F_c_ ^2^)^2^]/Σ[w(F_o_^2^) ^2^]}^1/2^

**Supplementary Table 3.** Bond lengths [Å] and angles [º] for **6aa**.

| C(1)-C(18) | 1.389(3) | C(10)-C(5)-Si(1) | 119.85(14) |
| --- | --- | --- | --- |
| C(1)-C(2) | 1.397(3) | C(7)-C(6)-C(5) | 120.87(18) |
| C(2)-C(3) | 1.388(3) | C(8)-C(7)-C(6) | 120.17(18) |
| C(3)-C(4) | 1.395(3) | C(9)-C(8)-C(7) | 119.86(17) |
| C(4)-C(17) | 1.399(3) | C(8)-C(9)-C(10) | 119.93(17) |
| C(5)-C(6) | 1.399(3) | C(9)-C(10)-C(5) | 121.02(18) |
| C(5)-C(10) | 1.405(3) | C(12)-C(11)-C(16) | 117.71(17) |
| C(5)-Si(1) | 1.8615(18) | C(12)-C(11)-Si(1) | 116.68(14) |
| C(6)-C(7) | 1.392(3) | C(16)-C(11)-Si(1) | 125.58(14) |
| C(7)-C(8) | 1.392(3) | C(13)-C(12)-C(11) | 121.53(18) |
| C(8)-C(9) | 1.389(3) | C(12)-C(13)-C(14) | 119.61(18) |
| C(9)-C(10) | 1.393(3) | C(15)-C(14)-C(13) | 120.26(17) |
| C(11)-C(12) | 1.406(3) | C(14)-C(15)-C(16) | 119.97(18) |
| C(11)-C(16) | 1.404(3) | C(15)-C(16)-C(11) | 120.92(17) |
| C(11)-Si(1) | 1.8664(18) | N(1)-C(17)-C(4) | 124.04(18) |
| C(12)-C(13) | 1.389(3) | N(1)-C(17)-C(18) | 116.97(17) |
| C(13)-C(14) | 1.391(3) | C(4)-C(17)-C(18) | 118.99(16) |
| C(14)-C(15) | 1.387(3) | C(1)-C(18)-C(17) | 120.91(18) |
| C(15)-C(16) | 1.398(3) | C(1)-C(18)-S(1) | 121.88(16) |
| C(17)-N(1) | 1.396(2) | C(17)-C(18)-S(1) | 117.21(13) |
| C(17)-C(18) | 1.406(3) | C(17)-N(1)-C(19) | 118.79(16) |
| C(18)-S(1) | 1.7773(19) | C(17)-N(1)-Si(1) | 117.18(13) |
| C(19)-N(1) | 1.459(3) | C(19)-N(1)-Si(1) | 122.22(13) |
| N(1)-Si(1) | 1.7288(17) | C(18)-S(1)-Si(1) | 92.22(6) |
| S(1)-Si(1) | 2.1536(7) | N(1)-Si(1)-C(5) | 113.18(8) |
| C(18)-C(1)-C(2) | 119.75(19) | N(1)-Si(1)-C(11) | 109.90(8) |
| C(3)-C(2)-C(1) | 119.54(18) | C(5)-Si(1)-C(11) | 112.74(8) |
| C(2)-C(3)-C(4) | 121.15(19) | N(1)-Si(1)-S(1) | 94.79(6) |
| C(3)-C(4)-C(17) | 119.63(19) | C(5)-Si(1)-S(1) | 112.05(6) |
| C(6)-C(5)-C(10) | 118.14(16) | C(11)-Si(1)-S(1) | 112.95(6) |
| C(6)-C(5)-Si(1) | 121.90(14) |  |  |

#

# 8. DFT calculations

**Computational Details**

All DFT calculations were carried out with Gaussian 16^13^ package. Geometry optimizations and vibrational frequencies of all the stationary points were performed using the M06-2X^14-16^ functional. Def2-SVP^17-18^ basis set was used for all atoms. To get more reliable energies, single point energies were computed at the M06-2X/Def2-TZVPP^17-18^ level for all the optimized structures. The solvent effect was treated with the SMD solvent model^19^ with chloroform as the solvent. The 3D structures were generated with CYL view package.^20^ Activation free energy barriers here are defined as the free energy difference between the transition state and the lowest-energy stationary point before it in the reaction pathways.

**Supplementary Fig. 12.** **3D structures involved in B(C_6_F_5_)_3_ catalyzed silane insertion into benzothiazole (distances are given in Å).** Color code: H, white; C, gray; B, pink; N, blue; S, yellow; F, green; Si, brown

# NMR spectra

3-methyl-2,2-diphenyl-2,3-dihydrobenzo[d][1,3,2]thiazasilole (**6aa**)

**Supplementary Fig. 13.** ^13^C NMR spectrum of **6aa** (126 MHz, RT, CDCl_3_)

**Supplementary Fig. 14.** ^1^H NMR spectrum of **6aa** (500 MHz, RT, CDCl_3_)

**Supplementary Fig. 15.** ^29^Si NMR spectrum of **6aa** (99 MHz, RT, CDCl_3_)

1. methoxy-3-methyl-2,2-diphenyl-2,3-dihydrobenzo[d][1,3,2]thiazasilole (**6ba**)

**Supplementary Fig. 16.** ^13^C NMR spectrum of **6ba** (126 MHz, RT, CDCl_3_)

**Supplementary Fig. 17.** ^1^H NMR spectrum of **6ba** (500 MHz, RT, CDCl_3_)

**Supplementary Fig. 18.** ^29^Si NMR spectrum of **6ba** (99 MHz, RT, CDCl_3_)

6-bromo-3-methyl-2,2-diphenyl-2,3-dihydrobenzo[d][1,3,2]thiazasilole (**6ca**)

**Supplementary Fig. 19.** ^13^C NMR spectrum of **6ca** (126 MHz, RT, CDCl_3_)

**Supplementary Fig. 20.** ^1^H NMR spectrum of **6ca** (500 MHz, RT, CDCl_3_)

**Supplementary Fig. 21.** ^29^Si NMR spectrum of **6ca** (99 MHz, RT, CDCl_3_)

5-bromo-3-methyl-2,2-diphenyl-2,3-dihydrobenzo[d][1,3,2]thiazasilole (**6da**)

**Supplementary Fig. 22.** ^13^C NMR spectrum of **6da** (126 MHz, RT, CDCl_3_)

**Supplementary Fig. 23.** ^1^H NMR spectrum of **6da** (500 MHz, RT, CDCl_3_)

**Supplementary Fig. 24.** ^29^Si NMR spectrum of **6da** (99 MHz, RT, CDCl_3_)

5-chloro-3-methyl-2,2-diphenyl-2,3-dihydrobenzo[d][1,3,2]thiazasilole (**6ea**)

**Supplementary Fig. 25.** ^13^C NMR spectrum of **6ea** (126 MHz, RT, CDCl_3_)

**Supplementary Fig. 26.** ^1^H NMR spectrum of **6ea** (500 MHz, RT, CDCl_3_)

**Supplementary Fig. 27.** ^29^Si NMR spectrum of **6ea** (99 MHz, RT, CDCl_3_)

3-methyl-2,2-diphenyl-5-(thiophen-2-yl)-2,3-dihydrobenzo[d][1,3,2]thiazasilole (**6fa**)

**Supplementary Fig. 28.** ^13^C NMR spectrum of **6fa** (126 MHz, RT, CDCl_3_)

**Supplementary Fig. 29.** ^1^H NMR spectrum of **6fa** (500 MHz, RT, CDCl_3_)

**Supplementary Fig. 30.** ^29^Si NMR spectrum of **6fa** (99 MHz, RT, CDCl_3_)

5-(benzo[b]thiophen-2-yl)-3-methyl-2,2-diphenyl-2,3-dihydrobenzo[d][1,3,2]thiazasilole (**6ga**)

**Supplementary Fig. 31.** ^13^C NMR spectrum of **6ga** (126 MHz, RT, CDCl_3_)

**Supplementary Fig. 32.** ^1^H NMR spectrum of **6ga** (500 MHz, RT, CDCl_3_)

**Supplementary Fig. 33.** ^29^Si NMR spectrum of **6ga** (99 MHz, RT, CDCl_3_)

5-([1,1'-biphenyl]-4-yl)-3-methyl-2,2-diphenyl-2,3-dihydrobenzo[d][1,3,2]thiazasilole (**6ha**)

**Supplementary Fig. 34.** ^13^C NMR spectrum of **6ha** (126 MHz, RT, CDCl_3_)

**Supplementary Figure. 35.** ^1^H NMR spectrum of **6ha** (500 MHz, RT, CDCl_3_)

**Supplementary Fig. 36.** ^29^Si NMR spectrum of **6ha** (99 MHz, RT, CDCl_3_)

3-methyl-6-(naphthalen-2-yl)-2,2-diphenyl-2,3-dihydrobenzo[d][1,3,2]thiazasilole (**6ia**)

**Supplementary Fig. 37.** ^13^C NMR spectrum of **6ia** (126 MHz, RT, CDCl_3_)

**Supplementary Fig. 38.** ^1^H NMR spectrum of **6ia** (500 MHz, RT, CDCl_3_)

**Supplementary Fig. 39.** ^29^Si NMR spectrum of **6ia** (99 MHz, RT, CDCl_3_)

3-methyl-2,2-diphenyl-6-(4,4,5,5-tetramethyl-1,3,2-dioxaborolan-2-yl)-2,3-dihydrobenzo

[d][1,3,2]thiazasilole (**6ja**)

**Supplementary Fig. 40.** ^13^C NMR spectrum of **6ja** (126 MHz, RT, CDCl_3_)

**Supplementary Fig. 41.** ^1^H NMR spectrum of **6ja** (500 MHz, RT, CDCl_3_)

**Supplementary Fig. 42.** ^29^Si NMR spectrum of **6ja** (99 MHz, RT, CDCl_3_)

6-(3,5-bis(trifluoromethyl)phenyl)-3-methyl-2,2-diphenyl-2,3-dihydrobenzo

[d][1,3,2]thiazasilole (**6ka**)

**Supplementary Fig. 43.** ^13^C NMR spectrum of **6ka** (126 MHz, RT, CDCl_3_)

**Supplementary Fig. 44.** ^1^H NMR spectrum of **6ka** (500 MHz, RT, CDCl_3_)

**Supplementary Figure. 45.** ^19^F NMR spectrum of **6ka** (471 MHz, RT, CDCl_3_)

**Supplementary Fig. 46.** ^29^Si NMR spectrum of **6ka** (99 MHz, RT, CDCl_3_)

3-ethyl-2,2-diphenyl-2,3-dihydrobenzo[d][1,3,2]thiazasilole (**6ma**)

**Supplementary Fig. 47.** ^13^C NMR spectrum of **6ma** (126 MHz, RT, CDCl_3_)

**Supplementary Fig. 48.** ^1^H NMR spectrum of **6ma** (500 MHz, RT, CDCl_3_)

**Supplementary Fig. 49.** ^29^Si NMR spectrum of **6ma** (99 MHz, RT, CDCl_3_)

5-chloro-3-ethyl-2,2-diphenyl-2,3-dihydrobenzo[d][1,3,2]thiazasilole (**6na**)

**Supplementary Fig. 50.** ^13^C NMR spectrum of **6na** (126 MHz, RT, CDCl_3_)

**Supplementary Fig. 51.** ^1^H NMR spectrum of **6na** (500 MHz, RT, CDCl_3_)

**Supplementary Fig. 52.** ^29^Si NMR spectrum of **6na** (99 MHz, RT, CDCl_3_)

3-ethyl-5,6-dimethyl-2,2-diphenyl-2,3-dihydrobenzo[d][1,3,2]thiazasilole (**6oa**)

**Supplementary Fig. 53.** ^13^C NMR spectrum of **6oa** (126 MHz, RT, CDCl_3_)

**Supplementary Fig. 54.** ^1^H NMR spectrum of **6oa** (500 MHz, RT, CDCl_3_)

**Supplementary Fig. 55.** ^29^Si NMR spectrum of **6oa** (99 MHz, RT, CDCl_3_)

1-ethyl-2,2-diphenyl-1,2-dihydronaphtho[1,2-d][1,3,2]thiazasilole (**6pa**)

**Supplementary Fig. 56.** ^13^C NMR spectrum of **6pa** (126 MHz, RT, CDCl_3_)

**Supplementary Fig. 57.** ^1^H NMR spectrum of **6pa** (500 MHz, RT, CDCl_3_)

**Supplementary Fig. 58.** ^29^Si NMR spectrum of **6pa** (99 MHz, RT, CDCl_3_)

2,2-diphenyl-3-propyl-2,3-dihydrobenzo[d][1,3,2]thiazasilole (**6qa**)

**Supplementary Fig. 59.** ^13^C NMR spectrum of **6qa** (126 MHz, RT, CDCl_3_)

**Supplementary Fig. 60.** ^1^H NMR spectrum of **6qa** (500 MHz, RT, CDCl_3_)

**Supplementary Fig. 61.** ^29^Si NMR spectrum of **6qa** (99 MHz, RT, CDCl_3_)

3-isobutyl-2,2-diphenyl-2,3-dihydrobenzo[d][1,3,2]thiazasilole (**6ra**)

**Supplementary Fig. 62.** ^13^C NMR spectrum of **6ra** (126 MHz, RT, CDCl_3_)

**Supplementary Fig. 63.** ^1^H NMR spectrum of **6ra** (500 MHz, RT, CDCl_3_)

**Supplementary Fig. 64.** ^29^Si NMR spectrum of **6ra** (99 MHz, RT, CDCl_3_)

3-(cyclopropylmethyl)-2,2-diphenyl-2,3-dihydrobenzo[d][1,3,2]thiazasilole (**6sa**)

**Supplementary Fig. 65.** ^13^C NMR spectrum of **6sa** (126 MHz, RT, CDCl_3_)

**Supplementary Fig. 66.** ^1^H NMR spectrum of **6sa** (500 MHz, RT, CDCl_3_)

**Supplementary Fig. 67.** ^29^Si NMR spectrum of **6sa** (99 MHz, RT, CDCl_3_)

3-(cyclohexylmethyl)-2,2-diphenyl-2,3-dihydrobenzo[d][1,3,2]thiazasilole (**6ta**)

**Supplementary Fig. 68.** ^13^C NMR spectrum of **6ta** (126 MHz, RT, CDCl_3_)

**Supplementary Fig. 69.** ^1^H NMR spectrum of **6ta** (500 MHz, RT, CDCl_3_)

**Supplementary Fig. 70.** ^29^Si NMR spectrum of **6ta** (99 MHz, RT, CDCl_3_)

2,2-diethyl-3-methyl-2,3-dihydrobenzo[d][1,3,2]thiazasilole (**6ab**)

**Supplementary Fig. 71.** ^13^C NMR spectrum of **6ab** (126 MHz, RT, CDCl_3_)

**Supplementary Fig. 72.** ^1^H NMR spectrum of **6ab** (500 MHz, RT, CDCl_3_)

**Supplementary Fig. 73.** ^29^Si NMR spectrum of **6ab** (99 MHz, RT, CDCl_3_)

2,3-dimethyl-2-phenyl-2,3-dihydrobenzo[d][1,3,2]thiazasilole (**6ac**)

**Supplementary Fig. 74.** ^13^C NMR spectrum of **6ac** (126 MHz, RT, CDCl_3_)

**Supplementary Fig. 75.** ^1^H NMR spectrum of **6ac** (500 MHz, RT, CDCl_3_)

**Supplementary Fig. 76.** ^29^Si NMR spectrum of **6ac** (99 MHz, RT, CDCl_3_)

3-methyl-2-phenyl-2,3-dihydrobenzo[d][1,3,2]thiazasilole (**6ad**)

**Supplementary Fig. 77.** ^13^C NMR spectrum of **6ad** (126 MHz, RT, CDCl_3_)

**Supplementary Fig. 78.** ^1^H NMR spectrum of **6ad** (500 MHz, RT, CDCl_3_)

**Supplementary Fig. 79.** ^29^Si NMR spectrum of **6ad** (99 MHz, RT, CDCl_3_)

4,4'-bis(3-methyl-2,3-dihydrobenzo[d][1,3,2]thiazasilol-2-yl)-1,1'-biphenyl (**6ae**)

**Supplementary Fig. 80.** ^13^C NMR spectrum of **6ae** (126 MHz, RT, C_6_D_6_)

**Supplementary Fig. 81.** ^1^H NMR spectrum of **6ae** (500 MHz, RT, C_6_D_6_)

**Supplementary Fig. 82.** ^29^Si NMR spectrum of **6ae** (99 MHz, RT, C_6_D_6_)

4,4'-bis(5-(benzo[b]thiophen-2-yl)-3-methyl-2,3-dihydrobenzo[d][1,3,2]thiazasilol-2-yl)

-1,1'-biphenyl (**6ge**)

**Supplementary Fig. 83.** ^13^C NMR spectrum of **6ge** (126 MHz, RT, C_6_D_6_)

**Supplementary Fig. 84.** ^1^H NMR spectrum of **6ge** (500 MHz, RT, C_6_D_6_)

**Supplementary Fig. 85.** ^29^Si NMR spectrum of **6ge** (99 MHz, RT, C_6_D_6_)

4,4'-bis(1-ethyl-1,2-dihydronaphtho[1,2-d][1,3,2]thiazasilol-2-yl)-1,1'-biphenyl (**6pe**)

**Supplementary Fig. 86.** ^13^C NMR spectrum of **6pe** (126 MHz, RT, C_6_D_6_)

**Supplementary Fig. 87.** ^1^H NMR spectrum of **6pe** (500 MHz, RT, C_6_D_6_)

**Supplementary Fig. 88.** ^29^Si NMR spectrum of **6pe** (99 MHz, RT, C_6_D_6_)

3-methyl-2,2-diphenyl-2,3-dihydrobenzo[d][1,3,2]oxazasilole (**7aa**)

**Supplementary Fig. 89.** ^13^C NMR spectrum of **7aa** (126 MHz, RT, CDCl_3_)

**Supplementary Fig. 90.** ^1^H NMR spectrum of **7aa** (500 MHz, RT, CDCl_3_)

**Supplementary Fig. 91.** ^29^Si NMR spectrum of **7aa** (99 MHz, RT, CDCl_3_)

3,5-dimethyl-2,2-diphenyl-2,3-dihydrobenzo[d][1,3,2]oxazasilole (**7ba**)

**Supplementary Fig. 92.** ^13^C NMR spectrum of **7ba** (126 MHz, RT, CDCl_3_)

**Supplementary Fig. 93.** ^1^H NMR spectrum of **7ba** (500 MHz, RT, CDCl_3_)

**Supplementary Fig. 94.** ^29^Si NMR spectrum of **7ba** (99 MHz, RT, CDCl_3_)

3,6-dimethyl-2,2-diphenyl-2,3-dihydrobenzo[d][1,3,2]oxazasilole (**7ca**)

**Supplementary Fig. 95.** ^13^C NMR spectrum of **7ca** (126 MHz, RT, CDCl_3_)

**Supplementary Fig. 96.** ^1^H NMR spectrum of **7ca** (500 MHz, RT, CDCl_3_)

**Supplementary Fig. 97.** ^29^Si NMR spectrum of **7ca** (99 MHz, RT, CDCl_3_)

5-chloro-3-methyl-2,2-diphenyl-2,3-dihydrobenzo[d][1,3,2]oxazasilole (**7da**)

**Supplementary Fig. 98.** ^13^C NMR spectrum of **7da** (126 MHz, RT, CDCl_3_)

**Supplementary Fig. 99.** ^1^H NMR spectrum of **7da** (500 MHz, RT, CDCl_3_)

**Supplementary Fig. 100.** ^29^Si NMR spectrum of **7da**(99 MHz, RT, CDCl_3_)

3-methyl-6-nitro-2,2-diphenyl-2,3-dihydrobenzo[d][1,3,2]oxazasilole (**7ea**)

**Supplementary Fig. 101.** ^13^C NMR spectrum of **7ea** (126 MHz, RT, CDCl_3_)

**Supplementary Fig. 102.** ^1^H NMR spectrum of **7ea** (500 MHz, RT, CDCl_3_)

**Supplementary Fig. 103.** ^29^Si NMR spectrum of **7ea** (99 MHz, RT, CDCl_3_)

3-ethyl-2,2-diphenyl-2,3-dihydrobenzo[d][1,3,2]oxazasilole (**7fa**)

**Supplementary Fig. 104.** ^13^C NMR spectrum of **7fa** (126 MHz, RT, CDCl_3_)

**Supplementary Fig. 105.** ^1^H NMR spectrum of **7fa** (500 MHz, RT, CDCl_3_)

**Supplementary Fig. 106.** ^29^Si NMR spectrum of **7fa** (99 MHz, RT, CDCl_3_)

3-ethyl-5-methyl-2,2-diphenyl-2,3-dihydrobenzo[d][1,3,2]oxazasilole (**7ga**)

**Supplementary Fig. 107.** ^13^C NMR spectrum of **7ga** (126 MHz, RT, CDCl_3_)

**Supplementary Fig. 108.** ^1^H NMR spectrum of **7ga** (500 MHz, RT, CDCl_3_)

**Supplementary Fig. 109.** ^29^Si NMR spectrum of **7ga** (99 MHz, RT, CDCl_3_)

5-chloro-3-ethyl-2,2-diphenyl-2,3-dihydrobenzo[d][1,3,2]oxazasilole (**7ha**)

**Supplementary Fig. 110.** ^13^C NMR spectrum of **7ha** (126 MHz, RT, CDCl_3_)

**Supplementary Fig. 111.** ^1^H NMR spectrum of **7ha** (500 MHz, RT, CDCl_3_)

**Supplementary Fig. 112.** ^29^Si NMR spectrum of **7ha** (99 MHz, RT, CDCl_3_)

3-ethyl-2,2,5-triphenyl-2,3-dihydrobenzo[d][1,3,2]oxazasilole (**7ia**)

**Supplementary Fig. 113.**^13^C NMR spectrum of **7ia** (126 MHz, RT, CDCl_3_)

**Supplementary Fig. 114.** ^1^H NMR spectrum of **7ia** (500 MHz, RT, CDCl_3_)

**Supplementary Fig. 115.** ^29^Si NMR spectrum of **7ia** (99 MHz, RT, CDCl_3_)

6-bromo-3-ethyl-2,2-diphenyl-2,3-dihydrobenzo[d][1,3,2]oxazasilole (**7ja**)

**Supplementary Fig. 116.** ^13^C NMR spectrum of **7ja** (126 MHz, RT, CDCl_3_)

**Supplementary Fig. 117.** ^1^H NMR spectrum of **7ja** (500 MHz, RT, CDCl_3_)

**Supplementary Fig. 118.** ^29^Si NMR spectrum of **7ja** (99 MHz, RT, CDCl_3_)

1-ethyl-2,2-diphenyl-1,2-dihydronaphtho[1,2-d][1,3,2]oxazasilole (**7ka**)

**Supplementary Fig. 119.** ^13^C NMR spectrum of **7ka** (126 MHz, RT, CDCl_3_)

**Supplementary Fig. 120.** ^1^H NMR spectrum of **7ka** (500 MHz, RT, CDCl_3_)

**Supplementary Fig. 121.** ^29^Si NMR spectrum of **7ka** (99 MHz, RT, CDCl_3_)

2,2-diethyl-3-methyl-2,3-dihydrobenzo[d][1,3,2]oxazasilole (**7ab**)

**Supplementary Fig. 122.** ^13^C NMR spectrum of **7ab** (126 MHz, RT, CDCl_3_)

**Supplementary Fig. 123.** ^1^H NMR spectrum of **7ab** (500 MHz, RT, CDCl_3_)

**Supplementary Fig. 124.** ^29^Si NMR spectrum of **7ab** (99 MHz, RT, CDCl_3_)

2,3-dimethyl-2-phenyl-2,3-dihydrobenzo[d][1,3,2]oxazasilole (**7ac**)

**Supplementary Fig. 125.** ^13^C NMR spectrum of **7ac** (126 MHz, RT, CDCl_3_)

**Supplementary Fig. 126.** ^1^H NMR spectrum of **7ac** (500 MHz, RT, CDCl_3_)

**Supplementary Fig. 127.** ^29^Si NMR spectrum of **7ac** (99 MHz, RT, CDCl_3_)

3-methyl-2-phenyl-2,3-dihydrobenzo[d][1,3,2]oxazasilole (**7ad**)

**Supplementary Fig. 128.** ^13^C NMR spectrum of **7ad** (126 MHz, RT, CDCl_3_)

**Supplementary Fig. 129.** ^1^H NMR spectrum of **7ad** (500 MHz, RT, CDCl_3_)

**Supplementary Fig. 130.** ^29^Si NMR spectrum of **7ad** (99 MHz, RT, CDCl_3_)

4,4'-bis(1-ethyl-1,2-dihydronaphtho[1,2-d][1,3,2]oxazasilol-2-yl)-1,1'-biphenyl (**7ke**)

**Supplementary Fig. 131.** ^13^C NMR spectrum of **7ke** (126 MHz, RT, C_6_D_6_)

**Supplementary Fig. 132.** ^1^H NMR spectrum of **7ke** (500 MHz, RT, C_6_D_6_)

**Supplementary Fig. 133.** ^29^Si NMR spectrum of **7ke** (99 MHz, RT, C_6_D_6_)

3-methyl-2,2-diphenyl-2,3-dihydrobenzo[d][1,3,2]selenazasilole (**8aa**)

**Supplementary Fig. 134.** ^13^C NMR spectrum of **8aa** (126 MHz, RT, CDCl_3_)

**Supplementary Fig. 135.** ^1^H NMR spectrum of **8aa** (500 MHz, RT, CDCl_3_)

**Supplementary Fig. 136.** ^29^Si NMR spectrum of **8aa** (99 MHz, RT, CDCl_3_)

3-ethyl-2,2-diphenyl-2,3-dihydrobenzo[d][1,3,2]selenazasilole (**8ba**)

**Supplementary Fig. 137.** ^13^C NMR spectrum of **8ba** (126 MHz, RT, CDCl_3_)

**Supplementary Fig. 138.** ^1^H NMR spectrum of **8ba** (500 MHz, RT, CDCl_3_)

**Supplementary Fig. 139.** ^29^Si NMR spectrum of **8ba** (99 MHz, RT, CDCl_3_)

2,2-diethyl-3-methyl-2,3-dihydrobenzo[d][1,3,2]selenazasilole (**8ab**)

**Supplementary Fig. 140.** ^13^C NMR spectrum of **8ab** (126 MHz, RT, CDCl_3_)

**Supplementary Fig. 141.** ^1^H NMR spectrum of **8ab** (500 MHz, RT, CDCl_3_)

**Supplementary Fig. 142.** ^29^Si NMR spectrum of **8ab** (99 MHz, RT, CDCl_3_)

2,3-dimethyl-2-phenyl-2,3-dihydrobenzo[d][1,3,2]selenazasilole (**8ac**)

**Supplementary Fig. 143.** ^13^C NMR spectrum of **8ac** (126 MHz, RT, CDCl_3_)

**Supplementary Fig. 144.** ^1^H NMR spectrum of **8ac** (500 MHz, RT, CDCl_3_)

**Supplementary Fig. 145.** ^29^Si NMR spectrum of **8ac** (99 MHz, RT, CDCl_3_)

3-methyl-2-phenyl-2,3-dihydrobenzo[d][1,3,2]selenazasilole (**8ad**)

**Supplementary Fig. 146.** ^13^C NMR spectrum of **8ad** (126 MHz, RT, CDCl_3_)

**Supplementary Fig. 147.** ^1^H NMR spectrum of **8ad** (500 MHz, RT, CDCl_3_)

**Supplementary Fig. 148.** ^29^Si NMR spectrum of **8ad** (99 MHz, RT, CDCl_3_)

4,4'-bis(3-methyl-2,3-dihydrobenzo[d][1,3,2]selenazasilol-2-yl)-1,1'-biphenyl (**8ae**)

**Supplementary Fig. 149.** ^13^C NMR spectrum of **8ae** (126 MHz, RT, C_6_D_6_)

**Supplementary Fig. 150.** ^1^H NMR spectrum of **8ae** (500 MHz, RT, C_6_D_6_)

**Supplementary Fig. 151.** ^29^Si NMR spectrum of **8ae** (99 MHz, RT, C_6_D_6_)

# References

1. Lehmann, M., Schulz, A. & Villinger, A. Bissilylated Halonium Ions: [Me_3_Si−X−SiMe_3_][B(C_6_F_5_)_4_] (X=F, Cl, Br, I). *Angew. Chem. Int. Ed.* **48**, 7444−7447 (2009).

2. Li, Z.-C. *et al.* Benzoselenazolium-based hemicyanine dye for G-Quadruplex detection. *Bioorg. Med. Chem. Lett.* **70**, 128801 (2022).

3. Karaj, E. *et al.* Tunable Cysteine-Targeting Electrophilic Heteroaromatic Warheads Induce Ferroptosis. *J. Med. Chem.* **65**, 11788-11817 (2022).

4. Guo, Y.-Q., Chen, F., Deng, C.-L. & Zhang, X.-G. Iodine-promoted ring-opening methylation of benzothiazoles with dimethyl sulfite. *Chem. Commun.* **57**, 1923−1926 (2021).

5. Babudri, F., Florio, S., Ronzini, L. & Aresta, M. Nickel-complex-catalyzed cross-coupling of grignard reagents with 2-halogenobenzothiazoles. *Tetrahedron* **39**, 1515−1521 (1983).

6. Minge, O., Mitzel, N. W. & Schmidbaur, H. Synthetic Pathways to Hydrogen-Rich Polysilylated Arenes from Trialkoxysilanes and Other Precursors. *Organometallics* **21**, 680−684 (2002).

7. Patil, S. S. & Bobade, V. D. Simple and Efficient One-Pot Synthesis of 2-Substituted Benzoxazole and Benzothiazole. *Synth. Commun.* **40**, 206−212 (2009).

8. Chulsky, K. & Dobrovetsky, R. B(C_6_F_5_)_3_-Catalyzed Selective Chlorination of Hydrosilanes. *Angew. Chem. Int. Ed.* **56**, 4744−4748 (2017).

9. Langer, R., Wünsche, L., Fenske, D. & Fuhr, O. Kupferchalkogenid-Clusterverbindungen mit Brom-funktionalisierter Ligandenhülle. *Z. Anorg. Allg. Chem.* **635**, 2488−2494 (2009).

10. Dhakshinamoorthy, A., Alvaro, M. & Garcia, H. Metal organic frameworks as heterogeneous catalysts for the selective N-methylation of aromatic primary amines with dimethyl carbonate. *Appl. Catal. A*. **378**, 19-25 (2010).

11. Dadwal, S., Kumar, M. & Bhalla, V. “Metal-Free” Nanoassemblies of AIEE-ICT-Active Pyrazine Derivative: Efficient Photoredox System for the Synthesis of Benzimidazoles. *J. Org. Chem.* **85**, 13906-13919 (2020).

12. Alaoui, A. E. *et al.* *Angew. Chem. Int. Ed.* **46**, 6469-6472 (2007).

13. Gaussian 16, R. A., Frisch, M. J. *et al.* Gaussian, Inc., Wallingford CT. Gaussian, Inc., Wallingford CT. (2016).

14. Zhao, Y., Schultz, N. E. & Truhlar, D. G. Design of Density Functionals by Combining the Method of Constraint Satisfaction with Parametrization for Thermochemistry, Thermochemical Kinetics, and Noncovalent Interactions. *J. Chem. Theory. Comput.* **2**, 364−382 (2006).

15. Zhao, Y. & Truhlar, D. G. A new local density functional for main-group thermochemistry, transition metal bonding, thermochemical kinetics, and noncovalent interactions. *J. Chem. Phys.* **125**, 194101 (2006).

16. Zhao, Y. & Truhlar, D. G. Density Functional for Spectroscopy: No Long-Range Self-Interaction Error, Good Performance for Rydberg and Charge-Transfer States, and Better Performance on Average than B_3_LYP for Ground States. *J. Phys. Chem. A.* **110**, 13126−13130 (2006).

17. Weigend, F. & Ahlrichs, R. Balanced basis sets of split valence, triple zeta valence and quadruple zeta valence quality for H to Rn: Design and assessment of accuracy. *Phys. Chem. Chem. Phys.* **7**, 3297−3305 (2005).

18. Weigend, F. Accurate Coulomb-fitting basis sets for H to Rn. *Phys. Chem. Chem. Phys.* **8**, 1057−1065 (2006).

19. Marenich, A. V, Cramer, C. J. & Truhlar, D. G. Universal Solvation Model Based on Solute Electron Density and on a Continuum Model of the Solvent Defined by the Bulk Dielectric Constant and Atomic Surface Tensions. *J. Phys. Chem. B.* **113**, 6378–6396 (2009).

20. Legault, C. Y. C. version 1.0b; Universitéde Sherbrooke: Quebec, Canada, <http://www.cylview.org> (2009).
