# Supplementary material for: Direct synthesis of sila-benzoazoles through hydrosilylation and rearrangement cascade reaction of benzoazoles and silanes
Source: Nat Commun. 2023 Feb 9;14:703. doi: 10.1038/s41467-023-36360-z (PMC9911738; doi:10.1038/s41467-023-36360-z)
Supplement: Supplementary file 3 — Description of Additional Supplementary Files [file 41467_2023_36360_MOESM3_ESM.pdf]

Description of Additional Supplementary Files

Supplementary Data 1: contains the calculated energies and Cartesian Coordinates of the optimized structures.
