# Supplementary material for: Direct synthesis of sila-benzoazoles through hydrosilylation and rearrangement cascade reaction of benzoazoles and silanes
Source: Nat Commun. 2023 Feb 9;14:703. doi: 10.1038/s41467-023-36360-z (PMC9911738; doi:10.1038/s41467-023-36360-z)
Supplement: Supplementary file 4 — Supplementary Data 1 [file 41467_2023_36360_MOESM4_ESM.docx]

**Energies and Cartesian Coordinates of the Optimized Structures**

**BCF**

M06-2X/Def2SVP Electronic E: -2205.887122 a.u.

M06-2X/Def2SVP Gibbs free E: -2205.787702 a.u.

M06-2X/Def2TZVPP Electronic E: -2208.480214 a.u.

B 0.0011052550 -0.0017870615 0.0010548331

C 0.0970284896 1.5679348209 0.0001229822

C -0.8516786201 2.3706567607 -0.6437918364

C 1.1357282286 2.2507838755 0.6432523189

C -0.7775270955 3.7576520143 -0.6655791584

C 1.2325713090 3.6365847824 0.6588505812

C 0.2701590182 4.3914967904 -0.0045668888

C 1.3122654577 -0.8702459381 -0.0011861109

C 2.4836460621 -0.4492030714 -0.6413314967

C 1.3822005902 -2.1134764484 0.6380431652

C 3.6486058218 -1.2057872065 -0.6590286886

C 2.5341732814 -2.8897089464 0.6565399784

C 3.6720153121 -2.4311428867 -0.0001067692

C -1.4072338031 -0.7018886231 0.0041901892

C -1.6332918904 -1.9218264626 -0.6434042082

C -2.5150754286 -0.1419327504 0.6507749154

C -2.8730315294 -2.5483406286 -0.6622286126

C -3.7649670404 -0.7478341824 0.6687475490

C -3.9424563988 -1.9565593386 0.0026930052

F 2.2247186092 4.2404285467 1.2916249086

F 0.3500096667 5.7062276391 -0.0061547433

F -1.6896860389 4.4760244150 -1.2990424354

F -1.8750826246 1.8193587531 -1.2851415769

F 2.0827599633 1.5806128095 1.2886676735

F 2.5193426538 0.7116583747 -1.2845348354

F 4.7287022751 -0.7739713284 -1.2886560734

F 4.7710260874 -3.1570847269 0.0010789491

F 2.5580174674 -4.0534213100 1.2848336130

F 0.3270013789 -2.6005961392 1.2797644830

F -0.6487461798 -2.5312549744 -1.2929536504

F -2.4037432681 1.0113938595 1.2989234439

F -4.7809880571 -0.1896064936 1.3056080784

F -5.1229554910 -2.5407290654 0.0025018174

F -3.0444804619 -3.6952048594 -1.2985084000

**Ph_2_SiH_2_**

M06-2X/Def2SVP Electronic E: -753.361455 a.u.

M06-2X/Def2SVP Gibbs free E: -753.201063 a.u.

M06-2X/Def2TZVPP Electronic E: -753.968843 a.u.

C 2.7376150000 1.4110630000 0.9636660000

C 1.5933210000 0.6171460000 0.8972640000

C 1.5433330000 -0.5101900000 0.0602890000

C 2.6741260000 -0.8148710000 -0.7126380000

C 3.8216650000 -0.0224230000 -0.6497960000

C 3.8543400000 1.0908280000 0.1895000000

H 2.7583020000 2.2835650000 1.6193910000

H 0.7237490000 0.8858050000 1.5045020000

H 2.6623010000 -1.6817160000 -1.3788160000

H 4.6912580000 -0.2736370000 -1.2600080000

H 4.7501560000 1.7128000000 0.2382920000

Si -0.0000070000 -1.5853820000 -0.0000280000

H -0.1000900000 -2.4596500000 1.2021450000

H 0.0998800000 -2.4597110000 -1.2021700000

C -1.5432550000 -0.5100490000 -0.0602580000

C -1.5932340000 0.6174400000 -0.8970390000

C -2.6741370000 -0.8149300000 0.7124690000

C -2.7375940000 1.4112550000 -0.9634810000

H -0.7235870000 0.8862910000 -1.5040910000

C -3.8217300000 -0.0225830000 0.6496070000

H -2.6622770000 -1.6818840000 1.3785140000

C -3.8543990000 1.0907950000 -0.1895240000

H -2.7582490000 2.2838620000 -1.6190680000

H -4.6913710000 -0.2739420000 1.2596930000

H -4.7502710000 1.7126840000 -0.2383340000

**Benzothiazole**

M06-2X/Def2SVP Electronic E: -722.200730 a.u.

M06-2X/Def2SVP Gibbs free E: -722.127520 a.u.

M06-2X/Def2TZVPP Electronic E: -722.697521 a.u.

C 0.5055323907 1.7901123800 -0.0683833711

C -0.9044471931 1.7171442806 -0.0921163904

C -1.6621330936 2.8953304385 0.0004829862

C -1.0016537534 4.1083597856 0.1136126958

C 0.4049395945 4.1671233403 0.1360649624

C 1.1732942724 3.0147251594 0.0458305857

C -0.4757085757 -0.4307255204 -0.2726743313

H -2.7508604658 2.8323913010 -0.0184063874

H -1.5773152053 5.0323095086 0.1864610574

H 0.9011720793 5.1347964639 0.2259252623

H 2.2628753762 3.0624575671 0.0634121506

H -0.6390850499 -1.5071016863 -0.3678312988

N -1.4205264911 0.4380535078 -0.2091659281

S 1.1614574047 0.1842375138 -0.1992376334

**Resting species**

M06-2X/Def2SVP Electronic E: -2928.146636 a.u.

M06-2X/Def2SVP Gibbs free E: -2927.948277 a.u.

M06-2X/Def2TZVPP Electronic E: -2931.225852 a.u.

C 0.8409584338 2.0252036985 -0.0730137666

C -0.5216876215 2.0547771962 -0.4026995394

C -1.0406871312 3.1165829463 -1.1581237423

C -0.1704758387 4.1200031238 -1.5517875829

C 1.1950436449 4.0843314353 -1.2135987982

C 1.7193965692 3.0371991790 -0.4730209574

C -0.4420693757 0.1340538962 0.7588712833

H -2.0928073676 3.1527861048 -1.4379700181

H -0.5546368212 4.9541530284 -2.1398380619

H 1.8516299088 4.8914667029 -1.5404017145

H 2.7765835901 3.0005765554 -0.2091120494

H -0.8066438020 -0.7678089777 1.2520568599

S 1.1945801247 0.5952359295 0.8583545029

B -2.8218057995 0.7860175718 -0.0268441723

C -3.3595059655 2.0538464110 0.8645965524

C -3.2990789416 0.7644178849 -1.5918644054

C -3.2704869853 -0.6855489075 0.5457266492

C -3.0565106083 2.0732924374 2.2263720774

C -4.0213595227 3.1853224464 0.3978793775

C -2.4958575187 0.5571172366 -2.7090922949

C -4.6711384577 0.7878797622 -1.8430224223

C -4.2746746560 -0.9303829405 1.4810383261

C -2.7292945516 -1.8276020749 -0.0482455826

C -3.4017639141 3.1084082622 3.0846946269

F -2.4110041984 1.0343244355 2.7684945162

C -4.3875374477 4.2508657033 1.2194970743

F -4.3201256981 3.3298048285 -0.8962865464

C -3.0082346454 0.4359421766 -4.0007534478

F -1.1697520303 0.4558085717 -2.5980373551

C -5.2251818197 0.6716061883 -3.1106728412

F -5.5171365187 0.9266346736 -0.8196361246

C -4.6921515708 -2.2151514465 1.8271263313

F -4.9078358272 0.0651702965 2.0962512983

C -3.1087278830 -3.1259175761 0.2711183880

F -1.7753767945 -1.6922970542 -0.9760368891

C -4.0755290004 4.2120809624 2.5711050678

F -3.0961611550 3.0581199830 4.3734310302

F -5.0158683002 5.3037295388 0.7157287896

C -4.3798998848 0.4944047419 -4.2022823909

F -2.1950524242 0.2508485297 -5.0322438769

F -6.5379580216 0.7152656663 -3.2899580423

C -4.1073111025 -3.3184375789 1.2194758110

F -5.6493032498 -2.3904799675 2.7264621266

F -2.5427046136 -4.1676347509 -0.3208664420

F -4.4109921082 5.2159088451 3.3643293568

F -4.8824107502 0.3771005891 -5.4211174844

F -4.4946944692 -4.5412387763 1.5403672447

N -1.2174280494 0.9431308316 0.0898346984

**Int1**

M06-2X/Def2SVP Electronic E: -3681.511478 a.u.

M06-2X/Def2SVP Gibbs free E: -3681.130613 a.u.

M06-2X/Def2TZVPP Electronic E: -3685.198723 a.u.

C 6.0976892064 0.0775501397 1.1636914554

C 4.7269848633 0.3762416521 1.2240715095

C 4.2763190626 1.5044021575 1.9250940470

C 5.2252947400 2.3006862491 2.5454379631

C 6.5975225187 1.9946936974 2.4807249249

C 7.0534244452 0.8814624341 1.7918470660

C 4.6680703696 -1.5010384411 -0.0197696969

H 3.2162339638 1.7521369259 1.9850088107

H 4.8993938319 3.1834480069 3.0963206028

H 7.3159210097 2.6448855545 2.9812182513

H 8.1155584827 0.6403326587 1.7392043590

H 4.2318792857 -2.3161354900 -0.6019973895

N 3.9468936644 -0.5577876313 0.5334019479

S 6.3463710955 -1.3726157407 0.2303461158

C 2.4921196354 2.1127112182 -2.9534157118

C 2.5145328374 0.9981201640 -2.1145439487

C 1.9866660128 1.0715762948 -0.8163954413

C 1.4483122071 2.2894292254 -0.3707073201

C 1.4475426340 3.4102229954 -1.1997213376

C 1.9624895161 3.3194096237 -2.4940284021

H 2.8862984853 2.0389785797 -3.9681840392

H 2.9457422698 0.0630865901 -2.4855684396

H 1.0108537250 2.3579166488 0.6299496012

H 1.0287379032 4.3524089723 -0.8408733275

H 1.9404200973 4.1905199710 -3.1521986386

Si 2.0463432146 -0.3946529666 0.3287468380

H 1.6924933942 -0.1224857674 1.7319567447

C 1.6380200494 -2.1097705124 -0.2671641190

C 1.7243737130 -3.1626255338 0.6617678628

C 1.2633349401 -2.4014898997 -1.5872333602

C 1.4450282551 -4.4728065134 0.2793506265

H 2.0006867518 -2.9605605177 1.7008500493

C 0.9701053818 -3.7129505457 -1.9660859044

H 1.1833255137 -1.6028644169 -2.3249508106

C 1.0592926107 -4.7471179092 -1.0344719483

H 1.5058250601 -5.2787572987 1.0121553903

H 0.6655218957 -3.9243927637 -2.9926219236

H 0.8178466760 -5.7702803071 -1.3293223406

H -0.2518030820 0.1052265581 0.3786693201

B -1.4611816208 0.1865488186 0.1321516354

C -1.8945497940 -1.3361748183 -0.2622273319

C -2.2089731504 0.6061223389 1.5169203361

C -1.5841904773 1.3038786702 -1.0516900658

C -2.5940187698 -1.7508602986 -1.3899370088

C -1.5069518554 -2.3591826370 0.6022805993

C -1.5408863005 1.0141898904 2.6632954579

C -3.5958294665 0.5526353506 1.6193512801

C -0.9795368196 1.0594138907 -2.2847034273

C -2.1106313203 2.5858249212 -0.9165693322

C -2.8525100784 -3.0906139933 -1.6776173477

F -3.0537496650 -0.8610220281 -2.2731389124

C -1.7583210883 -3.7057392098 0.3677584163

F -0.8377303039 -2.0599654928 1.7226073386

C -2.1934702881 1.3410684946 3.8506545003

F -0.2048007936 1.1222988955 2.6755339917

C -4.2924729309 0.8709744792 2.7793712073

F -4.3193020694 0.2075929112 0.5498840368

C -0.8785658144 1.9901454918 -3.3080200564

F -0.4302105097 -0.1376288578 -2.5186378160

C -2.0372986999 3.5568841641 -1.9173782267

F -2.7105451839 2.9789171992 0.2121738562

C -2.4296034564 -4.0737481311 -0.7925024590

F -3.5055028730 -3.4353191627 -2.7832178489

F -1.3591166461 -4.6408194666 1.2240611926

C -3.5790541624 1.2658980779 3.9072282600

F -1.5081715519 1.7248742478 4.9235213103

F -5.6184732030 0.8083235469 2.8265158685

C -1.4115578116 3.2601431939 -3.1183789516

F -0.2500635110 1.6988103662 -4.4422867064

F -2.5447769173 4.7695819112 -1.7184983388

F -2.6528863654 -5.3562415003 -1.0542538641

F -4.2197028828 1.5739193436 5.0276059887

F -1.2968038559 4.1819793308 -4.0665019685

**TS1/2**

M06-2X/Def2SVP Electronic E: -3681.493958 a.u.

M06-2X/Def2SVP Gibbs free E: -3681.114254 a.u.

M06-2X/Def2TZVPP Electronic E: -3685.177944 a.u.

C 6.2069501142 -0.7665824243 -1.9868783988

C 4.9150803057 -0.3455666604 -1.6794439469

C 4.6175469144 0.1895093412 -0.4151761825

C 5.6466551010 0.2921615901 0.5324967970

C 6.9422166934 -0.1267238482 0.2253040381

C 7.2217240072 -0.6562915033 -1.0337095626

H 6.4236246704 -1.1857031606 -2.9709008104

H 4.1287461305 -0.4516455022 -2.4333098097

H 5.4399056284 0.6998011404 1.5259695785

H 7.7332814380 -0.0423362547 0.9723817845

H 8.2334485676 -0.9880268487 -1.2739810367

Si 2.9117909954 0.8210255249 0.0053533248

H 2.7103398518 0.8552107972 1.4702254308

C 2.5219899556 2.4396133990 -0.8368337131

C 2.1974140762 3.5835072254 -0.0942477532

C 2.6085440634 2.5463465987 -2.2368011456

C 1.9430929732 4.7989346733 -0.7325705959

H 2.1266898939 3.5275952640 0.9933646929

C 2.3547087853 3.7579796488 -2.8751952422

H 2.8718798897 1.6742469565 -2.8436473050

C 2.0190120811 4.8852299002 -2.1216124219

H 1.6775264959 5.6758209413 -0.1398418393

H 2.4194052300 3.8249918903 -3.9624222527

H 1.8167588489 5.8340672297 -2.6217907853

H -0.5328064770 -0.0126735491 -0.3916668410

B -1.5540409990 -0.0025727805 0.4168765327

C -1.8393797706 1.5851082588 0.5678697802

C -2.6337168381 -0.8296515351 -0.4515685239

C -0.9434902569 -0.7603727327 1.7125026578

C -3.0195421702 2.2395929265 0.2232589473

C -0.8238958375 2.4159448500 1.0406823722

C -2.8321125163 -0.4848170888 -1.7864657902

C -3.3733531097 -1.9211221166 -0.0062723726

C -0.8281071907 -0.2377637171 2.9996311262

C -0.3923131721 -2.0320138398 1.5493906227

C -3.1883710299 3.6192221477 0.3428632668

F -4.0761887289 1.5657416951 -0.2321632005

C -0.9546984748 3.7884596162 1.2025821176

F 0.3446374038 1.8675081060 1.4024757419

C -3.6980810662 -1.1542435410 -2.6409179469

F -2.1562489708 0.5538522272 -2.3000863269

C -4.2520065332 -2.6271156423 -0.8256110903

F -3.2725724194 -2.3442721052 1.2544033913

C -0.1745027848 -0.9006876622 4.0370834514

F -1.3510818202 0.9491048036 3.3106270949

C 0.2744810600 -2.7285177018 2.5498753753

F -0.4944496714 -2.6438341651 0.3670602760

C -2.1495020661 4.3983457607 0.8320015103

F -4.3356222906 4.1897139617 -0.0008102474

F 0.0282686691 4.5260580920 1.7077428245

C -4.4148812064 -2.2407294728 -2.1503404001

F -3.8409040074 -0.7800820154 -3.9060849454

F -4.9367142562 -3.6609473955 -0.3530925359

C 0.3842031880 -2.1522673840 3.8107084601

F -0.0890270647 -0.3500315580 5.2416709340

F 0.7920960024 -3.9284515291 2.3186325656

F -2.2963049755 5.7077534117 0.9623156322

F -5.2458803126 -2.9032884568 -2.9410063462

F 1.0076116618 -2.7929651871 4.7881344512

C 1.2603035620 -2.4407812109 -1.7708152250

C 2.0182574538 -1.7903273514 -0.7876023777

C 2.8774198883 -2.5195164089 0.0378395122

C 2.9701399275 -3.8928174630 -0.1605500706

C 2.2288369170 -4.5336497941 -1.1624739547

C 1.3594250937 -3.8149549649 -1.9764886261

C 0.6756617522 -0.0670523495 -1.4500822962

H 3.4511254571 -2.0310766970 0.8266600110

H 3.6256848284 -4.4795584546 0.4838307556

H 2.3195627346 -5.6121969389 -1.2957470097

H 0.7581205870 -4.3124261178 -2.7383376139

H 0.4774872206 0.9773458000 -1.7024403399

N 1.7650332347 -0.4067878188 -0.7347998742

S 0.1805916644 -1.3088158300 -2.5604013004

**Int2**

M06-2X/Def2SVP Electronic E: -3681.493958 a.u.

M06-2X/Def2SVP Gibbs free E: -3681.114254 a.u.

M06-2X/Def2TZVPP Electronic E: -3685.177944 a.u.

C 6.2069501142 -0.7665824243 -1.9868783988

C 4.9150803057 -0.3455666604 -1.6794439469

C 4.6175469144 0.1895093412 -0.4151761825

C 5.6466551010 0.2921615901 0.5324967970

C 6.9422166934 -0.1267238482 0.2253040381

C 7.2217240072 -0.6562915033 -1.0337095626

H 6.4236246704 -1.1857031606 -2.9709008104

H 4.1287461305 -0.4516455022 -2.4333098097

H 5.4399056284 0.6998011404 1.5259695785

H 7.7332814380 -0.0423362547 0.9723817845

H 8.2334485676 -0.9880268487 -1.2739810367

Si 2.9117909954 0.8210255249 0.0053533248

H 2.7103398518 0.8552107972 1.4702254308

C 2.5219899556 2.4396133990 -0.8368337131

C 2.1974140762 3.5835072254 -0.0942477532

C 2.6085440634 2.5463465987 -2.2368011456

C 1.9430929732 4.7989346733 -0.7325705959

H 2.1266898939 3.5275952640 0.9933646929

C 2.3547087853 3.7579796488 -2.8751952422

H 2.8718798897 1.6742469565 -2.8436473050

C 2.0190120811 4.8852299002 -2.1216124219

H 1.6775264959 5.6758209413 -0.1398418393

H 2.4194052300 3.8249918903 -3.9624222527

H 1.8167588489 5.8340672297 -2.6217907853

H -0.5328064770 -0.0126735491 -0.3916668410

B -1.5540409990 -0.0025727805 0.4168765327

C -1.8393797706 1.5851082588 0.5678697802

C -2.6337168381 -0.8296515351 -0.4515685239

C -0.9434902569 -0.7603727327 1.7125026578

C -3.0195421702 2.2395929265 0.2232589473

C -0.8238958375 2.4159448500 1.0406823722

C -2.8321125163 -0.4848170888 -1.7864657902

C -3.3733531097 -1.9211221166 -0.0062723726

C -0.8281071907 -0.2377637171 2.9996311262

C -0.3923131721 -2.0320138398 1.5493906227

C -3.1883710299 3.6192221477 0.3428632668

F -4.0761887289 1.5657416951 -0.2321632005

C -0.9546984748 3.7884596162 1.2025821176

F 0.3446374038 1.8675081060 1.4024757419

C -3.6980810662 -1.1542435410 -2.6409179469

F -2.1562489708 0.5538522272 -2.3000863269

C -4.2520065332 -2.6271156423 -0.8256110903

F -3.2725724194 -2.3442721052 1.2544033913

C -0.1745027848 -0.9006876622 4.0370834514

F -1.3510818202 0.9491048036 3.3106270949

C 0.2744810600 -2.7285177018 2.5498753753

F -0.4944496714 -2.6438341651 0.3670602760

C -2.1495020661 4.3983457607 0.8320015103

F -4.3356222906 4.1897139617 -0.0008102474

F 0.0282686691 4.5260580920 1.7077428245

C -4.4148812064 -2.2407294728 -2.1503404001

F -3.8409040074 -0.7800820154 -3.9060849454

F -4.9367142562 -3.6609473955 -0.3530925359

C 0.3842031880 -2.1522673840 3.8107084601

F -0.0890270647 -0.3500315580 5.2416709340

F 0.7920960024 -3.9284515291 2.3186325656

F -2.2963049755 5.7077534117 0.9623156322

F -5.2458803126 -2.9032884568 -2.9410063462

F 1.0076116618 -2.7929651871 4.7881344512

C 1.2603035620 -2.4407812109 -1.7708152250

C 2.0182574538 -1.7903273514 -0.7876023777

C 2.8774198883 -2.5195164089 0.0378395122

C 2.9701399275 -3.8928174630 -0.1605500706

C 2.2288369170 -4.5336497941 -1.1624739547

C 1.3594250937 -3.8149549649 -1.9764886261

C 0.6756617522 -0.0670523495 -1.4500822962

H 3.4511254571 -2.0310766970 0.8266600110

H 3.6256848284 -4.4795584546 0.4838307556

H 2.3195627346 -5.6121969389 -1.2957470097

H 0.7581205870 -4.3124261178 -2.7383376139

H 0.4774872206 0.9773458000 -1.7024403399

N 1.7650332347 -0.4067878188 -0.7347998742

S 0.1805916644 -1.3088158300 -2.5604013004

**TS2/3**

M06-2X/Def2SVP Electronic E: -3681.499286 a.u.

M06-2X/Def2SVP Gibbs free E: -3681.113610 a.u.

M06-2X/Def2TZVPP Electronic E: -3685.178639 a.u.

C -3.9785309018 4.0031960430 1.0066519939

C -3.7689538828 2.6258904115 1.0799396545

C -4.1027194174 1.7903081762 0.0035175257

C -4.6939027775 2.3647366205 -1.1370173698

C -4.9016771169 3.7398127825 -1.2126575089

C -4.5370345438 4.5601607422 -0.1424919620

H -3.7060489081 4.6374711179 1.8515461871

H -3.3588881779 2.2039910586 1.9982141835

H -5.0052217057 1.7336514872 -1.9747635539

H -5.3563992662 4.1736568303 -2.1046558657

H -4.7005671627 5.6376186428 -0.2023153580

Si -3.8473571123 -0.0503536255 0.1103079739

H -3.4265584388 -0.5062576952 1.4532741620

C -5.3293662332 -0.9885747916 -0.5293141896

C -6.3522963538 -1.3298393061 0.3700877272

C -5.4884945396 -1.3070025249 -1.8870582872

C -7.5069722026 -1.9708032740 -0.0778973024

H -6.2518330943 -1.0975748266 1.4342556936

C -6.6435010987 -1.9468559348 -2.3354997361

H -4.7021093027 -1.0676353287 -2.6090768908

C -7.6527463410 -2.2785552261 -1.4308131903

H -8.2935198536 -2.2330895593 0.6315414077

H -6.7541813278 -2.1913614216 -3.3932442413

H -8.5549205112 -2.7829614886 -1.7813250501

B 1.6318073340 0.0146564958 -0.2414332045

C 0.5665639439 0.3360427340 0.9556381677

C 2.6767740652 -1.2157915261 0.0398481328

C 2.5937526679 1.2903490104 -0.6168915400

C 0.1590381076 1.6317481355 1.2774912365

C -0.0801509284 -0.6720899948 1.6766229641

C 3.4353209072 -1.7318334224 -1.0142940670

C 3.0145722980 -1.7180756920 1.2975520269

C 2.7732799505 1.9187096148 -1.8444974682

C 3.4084435089 1.7706715163 0.4109054569

C -0.7701710835 1.9199353798 2.2741404343

F 0.6457667361 2.6914395009 0.6291496744

C -1.0328959625 -0.4287591436 2.6593258152

F 0.1986042524 -1.9563713762 1.4533345971

C 4.3902201383 -2.7320924574 -0.8654296106

F 3.2623601458 -1.2701125249 -2.2538596398

C 3.9659787861 -2.7184059267 1.4914985645

F 2.4525922228 -1.2459868567 2.4090730785

C 3.6848920786 2.9564432208 -2.0446493023

F 2.0676120547 1.5616189307 -2.9189097496

C 4.3245113787 2.8028805984 0.2591672478

F 3.3093287985 1.2315991071 1.6292302948

C -1.3754817120 0.8808793001 2.9653255755

F -1.1205593149 3.1742851128 2.5329045745

F -1.6490072916 -1.4313361874 3.2728786790

C 4.6579325737 -3.2314205569 0.4032834433

F 5.0561026538 -3.1960928595 -1.9157887357

F 4.2330837246 -3.1654854321 2.7122095028

C 4.4653691898 3.4018220978 -0.9885241360

F 3.8085064451 3.5182924517 -3.2410911982

F 5.0619130428 3.2181689148 1.2810690578

F -2.3237959187 1.1350302003 3.8580177536

F 5.5683665553 -4.1782395743 0.5723198446

F 5.3334673622 4.3871279706 -1.1632088554

C -0.4478836457 -1.8247490255 -1.3097516149

C -1.8252170822 -1.7800088410 -0.9821889054

C -2.4891651634 -2.9196431766 -0.5146200967

C -1.8050111052 -4.1241508066 -0.4062761086

C -0.4608385081 -4.1980695916 -0.7748950207

C 0.2044599269 -3.0624077803 -1.2204182611

C -2.1109974308 0.2703397478 -2.0328575375

H -3.5497394839 -2.8753995544 -0.2629689696

H -2.3311479033 -5.0106167264 -0.0506685711

H 0.0726505407 -5.1478138972 -0.7178375956

H 1.2522834830 -3.1260492366 -1.5137799306

H -1.7141150086 -0.1480378548 -2.9573150982

N -2.4865994025 -0.5170185173 -1.0609247724

S 0.4474778417 -0.3773099795 -1.8178257242

H -2.3972774990 1.3262585686 -2.0118004116

**Int3**

M06-2X/Def2SVP Electronic E: -3681.499800 a.u.

M06-2X/Def2SVP Gibbs free E: -3681.115600 a.u.

M06-2X/Def2TZVPP Electronic E: -3685.179389 a.u.

C -4.2062856652 4.0244109765 0.8326755462

C -3.9464886515 2.6582544996 0.9362217959

C -4.4068043936 1.7640900890 -0.0422995681

C -5.1687966390 2.2675641162 -1.1123561501

C -5.4272758213 3.6323577120 -1.2180050677

C -4.9401976807 4.5113813836 -0.2482152162

H -3.8353729242 4.7049622781 1.6005772831

H -3.3971851298 2.2906746690 1.8030396052

H -5.5721057857 1.5884975230 -1.8691785642

H -6.0164889836 4.0116625918 -2.0543897872

H -5.1437061110 5.5804550359 -0.3314245937

Si -4.0626375316 -0.0575183230 0.0933968514

H -3.5547803157 -0.4731104294 1.4174036718

C -5.5030819835 -1.0909749460 -0.4864326871

C -6.4145946379 -1.5688634320 0.4688329160

C -5.7401445234 -1.3623662002 -1.8433964599

C -7.5376087111 -2.2968574635 0.0762191906

H -6.2498519909 -1.3779152225 1.5329308495

C -6.8631371368 -2.0892717882 -2.2357052440

H -5.0421368394 -1.0166930439 -2.6114786494

C -7.7618895991 -2.5565186915 -1.2756617970

H -8.2368633632 -2.6652038787 0.8285943384

H -7.0350272327 -2.2960782834 -3.2932599755

H -8.6387676025 -3.1290884880 -1.5829106651

B 1.5507954250 0.0298109343 -0.2867507128

C 0.4847631274 0.3636359363 0.9084573827

C 2.5762215034 -1.2166834510 0.0042696139

C 2.5447828521 1.2884460890 -0.6422924518

C 0.0824078970 1.6633116050 1.2201820252

C -0.1843664252 -0.6359671682 1.6202138953

C 3.3328876368 -1.7476309257 -1.0438658285

C 2.9001201519 -1.7206452972 1.2648706273

C 2.7783749043 1.8925752375 -1.8734491382

C 3.3371900834 1.7683490376 0.4031215006

C -0.8669637653 1.9620911529 2.1941734128

F 0.5919382391 2.7169347308 0.5789593848

C -1.1547644191 -0.3820344286 2.5831467346

F 0.0845469240 -1.9238385657 1.4045152419

C 4.2708445909 -2.7627123277 -0.8866038040

F 3.1758849660 -1.2869704549 -2.2855250189

C 3.8348266514 -2.7350659290 1.4675320639

F 2.3382486542 -1.2386052606 2.3727933845

C 3.7163440213 2.9097151118 -2.0588654572

F 2.1064530755 1.5302556933 -2.9670384948

C 4.2783374241 2.7797928787 0.2658765956

F 3.1935357218 1.2477043752 1.6251784879

C -1.4962400614 0.9306937174 2.8758212798

F -1.2188896734 3.2202455255 2.4368168897

F -1.7909745647 -1.3787188327 3.1871621056

C 4.5243858036 -3.2623938777 0.3848758748

F 4.9352346873 -3.2407576907 -1.9320594197

F 4.0877212110 -3.1829284536 2.6914316962

C 4.4709953585 3.3568714153 -0.9851077695

F 3.8902168927 3.4499106790 -3.2594090329

F 4.9916567648 3.1956799351 1.3050182877

F -2.4642765762 1.1955326116 3.7443681166

F 5.4191358260 -4.2232893154 0.5615924459

F 5.3643470596 4.3221668181 -1.1463709429

C -0.5753385304 -1.7261493693 -1.3612103603

C -1.9485958246 -1.6940765208 -1.0172228565

C -2.5950742421 -2.8392069511 -0.5348086357

C -1.9087891792 -4.0424210939 -0.4364351333

C -0.5736067645 -4.1105858924 -0.8348250190

C 0.0736111781 -2.9693751679 -1.2895635985

C -2.4501504709 0.3174104378 -2.1186529722

H -3.6514162483 -2.8041842520 -0.2664029806

H -2.4267784319 -4.9287466250 -0.0690440837

H -0.0330930542 -5.0571529554 -0.7913135267

H 1.1156425801 -3.0247971291 -1.6040963049

H -1.8757263177 -0.0315618856 -2.9765306244

N -2.6883366211 -0.4657923228 -1.1201623232

S 0.3613009463 -0.3022576436 -1.8648891967

H -2.8984557342 1.3155251812 -2.1436109831

**Int4**

M06-2X/Def2SVP Electronic E: -3681.505984 a.u.

M06-2X/Def2SVP Gibbs free E: -3681.121088 a.u.

M06-2X/Def2TZVPP Electronic E: -3685.183726 a.u.

C 6.0655750271 -2.6708309422 -2.5010986102

C 4.7982934873 -2.3022588100 -2.0456612525

C 4.5396666281 -0.9871260459 -1.6251463772

C 5.5867352641 -0.0503575864 -1.6651861512

C 6.8515401531 -0.4161797569 -2.1200298072

C 7.0908421484 -1.7272354362 -2.5393143860

H 6.2513132199 -3.6968685912 -2.8229760528

H 4.0030520617 -3.0533315743 -2.0150562222

H 5.4215395273 0.9771620318 -1.3301982657

H 7.6566793593 0.3202270731 -2.1429990633

H 8.0830115839 -2.0134705575 -2.8929607335

Si 2.8153245968 -0.5711991380 -1.0284527155

H 2.0680301699 -1.8391414117 -0.9937130907

C 2.7540414763 0.4852506068 0.4964477389

C 2.2387613585 -0.0468689998 1.6884723617

C 3.2474566040 1.7995860316 0.4975196351

C 2.2267581364 0.7142884819 2.8561535393

H 1.8282121222 -1.0599390519 1.7032735023

C 3.2271822698 2.5642955282 1.6629646246

H 3.6383692575 2.2456582136 -0.4216462984

C 2.7194224642 2.0196068033 2.8429075681

H 1.8218594621 0.2908887077 3.7767186495

H 3.6051632530 3.5878508656 1.6494706153

H 2.7017412086 2.6180300843 3.7555325834

B -1.7267107238 -0.8676621562 -1.9994301407

C -0.9531318358 -2.1119152838 -2.7333233480

C -2.8510526196 -1.3606890014 -0.9191308783

C -2.1983317045 0.3239285811 -3.0106719728

C -0.1537721181 -2.9723962625 -1.9792752076

C -0.9756906616 -2.3936454645 -4.0967202882

C -3.3133439513 -2.6705437640 -0.8012341402

C -3.3830543920 -0.4715793495 0.0213793621

C -3.4222738217 0.9923021706 -2.9834391508

C -1.2913990347 0.8489600646 -3.9339914569

C 0.6605277167 -3.9551825658 -2.5267251604

F -0.1397159116 -2.8714954562 -0.6472457509

C -0.1910122399 -3.3831626922 -4.6899032790

F -1.7539933702 -1.7013642452 -4.9315197797

C -4.2083442374 -3.0859643048 0.1856768258

F -2.9221322436 -3.6269364899 -1.6466135577

C -4.2754415714 -0.8420715749 1.0183129108

F -3.0678313958 0.8236210387 -0.0231254788

C -3.6891107943 2.1371571957 -3.7383293537

F -4.4359757930 0.5698069139 -2.2291790254

C -1.5050516475 1.9842344005 -4.7002391530

F -0.1042484846 0.2517487866 -4.1065916646

C 0.6418375521 -4.1644836822 -3.9014090901

F 1.4550303235 -4.6879513154 -1.7530144676

F -0.2366357688 -3.5835993949 -6.0020383566

C -4.6924998964 -2.1664842640 1.1025402307

F -4.6042552383 -4.3520755587 0.2445273627

F -4.7455438251 0.0535627763 1.8784428485

C -2.7253140392 2.6432105440 -4.5977122553

F -4.8711789124 2.7337578339 -3.6504603453

F -0.5635543793 2.4360070326 -5.5222608699

F 1.4065795544 -5.0988501160 -4.4480793614

F -5.5477224133 -2.5398417434 2.0426638748

F -2.9670256476 3.7279068157 -5.3173937567

C 0.1500316163 1.5282327253 -1.4345143030

C 1.3197398106 1.6777751220 -2.1965411440

C 1.6771834553 2.9077081303 -2.7522445476

C 0.8560834095 4.0149464713 -2.5548240186

C -0.3050508026 3.8901396674 -1.7909455295

C -0.6475709595 2.6610459436 -1.2315895786

C 2.5427555890 0.2863450553 -3.6414545876

H 2.6056721999 3.0003737251 -3.3196881711

H 1.1339150183 4.9767221237 -2.9875149358

H -0.9467219434 4.7570640612 -1.6257076362

H -1.5558094146 2.5513923210 -0.6376601618

H 2.1662882839 0.8928647779 -4.4707235868

N 2.1726766041 0.5351602964 -2.4413826399

S -0.2896048538 -0.0464267373 -0.7673020810

H 3.2330416735 -0.5404214174 -3.8385993164

**TS4/5**

M06-2X/Def2SVP Electronic E: -3681.494140 a.u.

M06-2X/Def2SVP Gibbs free E: -3681.106008 a.u.

M06-2X/Def2TZVPP Electronic E: -3685.170130 a.u.

Si -2.4515649543 0.0173203309 -1.3746716525

C -3.9796892865 -0.7539480465 -0.5715698951

C -5.1792885317 -0.0676139986 -0.3130842058

C -3.9439215970 -2.1332044724 -0.3225447995

C -6.2912867022 -0.7356654728 0.1969733111

H -5.2532704940 1.0049338769 -0.5150225643

C -5.0435085591 -2.8039447264 0.2163870366

H -3.0453498383 -2.7021289294 -0.5714116275

C -6.2197385314 -2.1030389324 0.4766083025

H -7.2185222573 -0.1893138650 0.3791025822

H -4.9797019580 -3.8745297572 0.4199950254

H -7.0875791151 -2.6221736094 0.8875832083

B 1.2283323327 -0.5323997990 0.4609477822

C 1.7607412902 0.7813148723 1.2446900198

C 0.2773008293 -1.5329425928 1.3128114801

C 2.3118718602 -1.3097710149 -0.4736061312

C 0.8655033465 1.5482228754 1.9949914358

C 3.0472628218 1.3160892925 1.1686449462

C -0.4888397869 -2.4978466060 0.6598470545

C 0.2374769967 -1.5997235575 2.7045749474

C 2.6617779815 -2.6514783524 -0.3137588617

C 2.9621609649 -0.6635621658 -1.5296019285

C 1.1604276603 2.7813228651 2.5542713548

F -0.3822055981 1.1070333335 2.1785164048

C 3.3957354602 2.5479836166 1.7264678073

F 4.0383679973 0.6725431633 0.5566757682

C -1.2646801311 -3.4469850442 1.3132024092

F -0.4576917568 -2.5630720789 -0.6752630377

C -0.5456440349 -2.5184971399 3.4018913630

F 0.9781623091 -0.7879529199 3.4578760491

C 3.5547278535 -3.3167299848 -1.1538809019

F 2.1631529923 -3.3899428672 0.6784524911

C 3.8556702931 -1.2868601465 -2.3895384378

F 2.7636776743 0.6393945563 -1.7379584052

C 2.4477101955 3.2899012173 2.4157085745

F 0.2339407832 3.4682335454 3.2103373784

F 4.6341754710 3.0070308371 1.6099022068

C -1.3084112568 -3.4437974942 2.7027747628

F -1.9580751434 -4.3512206312 0.6320481520

F -0.5543713314 -2.5234293695 4.7278136498

C 4.1537187339 -2.6321658111 -2.2002702311

F 3.8409130269 -4.5953012822 -0.9493159288

F 4.4362540332 -0.6119353256 -3.3720807363

F 2.7641276231 4.4619617571 2.9394040074

F -2.0494101468 -4.3275971617 3.3496663750

F 5.0065328277 -3.2463823886 -3.0030539381

C -0.1753325386 2.0563740126 -0.7237135215

C -1.3455330620 2.4740592495 -0.0761531681

C -1.5190363238 3.8070560824 0.2933033234

C -0.5058065266 4.7246641007 0.0221042161

C 0.6560148432 4.3167882952 -0.6362229865

C 0.8217069441 2.9862470955 -1.0194401970

C -2.9247896705 1.4321104514 1.2879482621

H -2.4446445855 4.1243247095 0.7773029183

H -0.6330826993 5.7682062854 0.3128955133

H 1.4403624124 5.0420092834 -0.8580605614

H 1.7273949056 2.6614486870 -1.5310543595

H -2.6938587850 2.1637949553 2.0718690955

N -2.3243460720 1.4691319357 0.1657043209

S -0.0482043856 0.3317476089 -1.0878250582

H -3.6609161069 0.6490993861 1.4860446973

C -2.7697932519 1.3070132842 -2.7012332932

C -2.2581897990 1.0738154749 -3.9870756185

C -3.5172728003 2.4754078157 -2.4846561184

C -2.4872098305 1.9771285024 -5.0253990920

H -1.6681847415 0.1739559682 -4.1809361007

C -3.7555632435 3.3771546931 -3.5207717904

H -3.9196315232 2.6947225219 -1.4918773440

C -3.2382333517 3.1289553410 -4.7931085401

H -2.0776289035 1.7807749754 -6.0178187920

H -4.3428852624 4.2781326166 -3.3348830769

H -3.4193915568 3.8367857016 -5.6041375235

H -1.9965204323 -1.1980876295 -2.1201478078

**Int5**

M06-2X/Def2SVP Electronic E: -1475.583416 a.u.

M06-2X/Def2SVP Gibbs free E: -1475.325982 a.u.

M06-2X/Def2TZVPP Electronic E: -1476.682197 a.u.

C 2.1753338260 -1.6078578879 -2.4559323801

C 2.0292445023 -0.9235964624 -1.2484251325

C 2.8199956468 -1.2480244584 -0.1363311841

C 3.7580321607 -2.2838544597 -0.2655947005

C 3.9147977656 -2.9640480953 -1.4742851265

C 3.1221817786 -2.6264321942 -2.5717954793

H 1.5469298692 -1.3456222728 -3.3092836878

H 1.2823059333 -0.1275164182 -1.1726220156

H 4.3751185504 -2.5731888165 0.5908272845

H 4.6530888763 -3.7640463184 -1.5576310732

H 3.2391254406 -3.1606640823 -3.5166079089

Si 2.6403123264 -0.3720159323 1.5249205176

C 1.0193218903 2.1615161396 0.5653063194

C 2.2601393448 2.4086314067 -0.0496732687

C 2.3826699528 3.4217263169 -1.0072359928

C 1.2856928788 4.2140916995 -1.3278448487

C 0.0591976204 3.9903266870 -0.6965683739

C -0.0768916518 2.9685140042 0.2391241885

C 4.5337212570 1.7956327825 0.1611744638

H 3.3367347592 3.5790472711 -1.5128182731

H 1.3840315443 5.0008657949 -2.0767706226

H -0.8045426188 4.6088342211 -0.9459422262

H -1.0403586684 2.7843482429 0.7174967039

H 4.9049206632 2.7124410196 -0.3183932205

N 3.3045007263 1.5525897740 0.3458736748

S 0.8300602604 0.8503745130 1.7384889314

H 5.2681585251 1.0573690181 0.5085305028

C 2.0283122351 -1.7445898640 2.7075305017

C 2.5918675740 -1.8870942567 3.9837864252

C 1.0008193300 -2.6312356367 2.3427545402

C 2.1497178410 -2.8743874466 4.8680487112

H 3.3957199350 -1.2140802466 4.2982158061

C 0.5523920064 -3.6192927056 3.2183485714

H 0.5422100420 -2.5501294878 1.3520754003

C 1.1276710431 -3.7420258382 4.4853740392

H 2.6041971214 -2.9665996796 5.8566815556

H -0.2466934333 -4.2982254921 2.9135633232

H 0.7790732264 -4.5157601421 5.1723652573

H 3.9091893091 0.0054137537 2.2053537167

**Int6**

M06-2X/Def2SVP Electronic E: -3681.501895 a.u.

M06-2X/Def2SVP Gibbs free E: -3681.114870 a.u.

M06-2X/Def2TZVPP Electronic E: -3685.175716 a.u.

C -2.3350526042 3.9489468593 3.7169500679

C -2.2011360264 3.2636005515 2.5108218826

C -0.9600079340 2.7378895141 2.1140570183

C 0.1420491471 2.9094510028 2.9659226550

C 0.0053554876 3.5849734242 4.1800632082

C -1.2311899361 4.1073944607 4.5560451147

H -3.3054849158 4.3581653019 4.0033386371

H -3.0789519730 3.1397475750 1.8698843799

H 1.1191179038 2.5094986978 2.6913417828

H 0.8720573422 3.7029335009 4.8327689659

H -1.3357349165 4.6385592225 5.5038355992

Si -0.8615165433 1.8665411255 0.4503753958

C -3.6819279901 0.7909758759 -0.3680527895

C -3.4058569859 0.0187676887 0.7680569913

C -4.2959222301 -0.9891159423 1.1601687335

C -5.4545508271 -1.2165390257 0.4278139576

C -5.7322157947 -0.4405801089 -0.7021308206

C -4.8511578235 0.5585961215 -1.1019520584

C -1.8718642407 -0.1780360774 2.5506655630

H -4.0672754694 -1.6304457088 2.0124058740

H -6.1352323193 -2.0134089608 0.7292086379

H -6.6360803372 -0.6256818652 -1.2842166216

H -5.0560584159 1.1533130403 -1.9937541125

H -2.5371737944 -0.8417287101 3.1165172663

N -2.1805029849 0.3025936954 1.4143481462

S -2.5339204148 2.0123202088 -0.9221730440

H -0.9142185201 0.1016288401 2.9981689587

C 0.3445286595 2.8908265236 -0.6290009958

C 0.3691907228 2.7633248323 -2.0302533967

C 1.2573587927 3.7899429942 -0.0497061930

C 1.2799379953 3.4736419396 -2.8125230392

H -0.3295706662 2.0945006291 -2.5299668908

C 2.1728834539 4.5003889257 -0.8267152434

H 1.2693034300 3.9478788229 1.0285268011

C 2.1951884825 4.3369352412 -2.2110634585

H 1.2747021492 3.3457349216 -3.8968536969

H 2.8753127973 5.1819289510 -0.3436216915

H 2.9183813909 4.8847222779 -2.8184426362

H 0.0915910499 0.5068498079 0.4576027891

B 0.5918695478 -0.6721646527 -0.1276567327

C 1.7608669189 -0.1051692173 -1.0796513664

C 1.2738081346 -1.4691200154 1.1083456837

C -0.7222723192 -1.3527579145 -0.7727243938

C 2.0350454222 -0.5305150024 -2.3772512036

C 2.6349522160 0.8520447270 -0.5622763672

C 1.5602751549 -0.9951426753 2.3804385759

C 1.7320896604 -2.7583602176 0.8353617834

C -1.2685470632 -0.9404635234 -1.9915210728

C -1.4679562728 -2.3300626213 -0.1075989395

C 3.0494503014 0.0288086120 -3.1512445538

F 1.3361722431 -1.5164670739 -2.9349874942

C 3.6565013985 1.4368232715 -1.2983890378

F 2.4907094768 1.2535357686 0.7032700218

C 2.2318611433 -1.7487684925 3.3421975887

F 1.1859263320 0.2322857320 2.7558193300

C 2.4059645497 -3.5454402278 1.7593897627

F 1.5100460434 -3.2848080339 -0.3703875473

C -2.4594746566 -1.4333982004 -2.5132641866

F -0.6725649657 0.0060128414 -2.7124633905

C -2.6485397441 -2.8695001813 -0.6068769733

F -1.1205012648 -2.7554412334 1.1096064702

C 3.8559282081 1.0241150408 -2.6112756157

F 3.2640146555 -0.3923011936 -4.3902910869

F 4.4382174061 2.3666600931 -0.7689765623

C 2.6591954650 -3.0312855710 3.0281360643

F 2.4684346586 -1.2468216248 4.5461372517

F 2.8115966640 -4.7673956528 1.4478193714

C -3.1598176260 -2.4046426705 -1.8102403237

F -2.9541508394 -0.9484906350 -3.6421018836

F -3.3377649719 -3.7504181648 0.1055583967

F 4.8174198881 1.5689184818 -3.3385317139

F 3.3004860861 -3.7608926592 3.9261962711

F -4.3164719912 -2.8559632884 -2.2614528632

**TS6/7**

M06-2X/Def2SVP Electronic E: -3681.499481 a.u.

M06-2X/Def2SVP Gibbs free E: -3681.109908 a.u.

M06-2X/Def2TZVPP Electronic E: -3685.176517 a.u.

C 2.9050721753 -3.9624844059 3.5325448248

C 2.6906752509 -3.3112429213 2.3178909255

C 1.4453464462 -2.7319120722 2.0226486600

C 0.4250009327 -2.8110108506 2.9858088523

C 0.6434699363 -3.4550910306 4.2038351834

C 1.8823062349 -4.0344253607 4.4777414307

H 3.8762482142 -4.4153569759 3.7396942076

H 3.5046017951 -3.2638820430 1.5897302586

H -0.5520404958 -2.3627587151 2.7960075958

H -0.1610066705 -3.5052607446 4.9395733030

H 2.0506501905 -4.5424943725 5.4289240534

Si 1.2415448682 -1.8450334661 0.3772019884

C 3.8352396681 -0.6172350555 -0.5612838721

C 3.3878072444 0.2049558916 0.4805399640

C 4.0858252956 1.3723006820 0.8085037351

C 5.2336732355 1.7106064408 0.1019443826

C 5.6783183874 0.8934272852 -0.9411215412

C 4.9853328786 -0.2663378657 -1.2741010724

C 1.7684159432 0.3235886545 2.2064796223

H 3.7191771588 2.0443153743 1.5854683871

H 5.7662948446 2.6292546856 0.3494955534

H 6.5692578938 1.1680598612 -1.5075488403

H 5.3240880096 -0.8986325310 -2.0958769360

H 2.3457861324 1.0908077709 2.7322579933

N 2.1764292504 -0.1921565872 1.1128818535

S 2.8927740842 -2.0362519279 -1.0170070191

H 0.8218024128 -0.0136667830 2.6371699683

C -0.0787320728 -2.7585667372 -0.6287815381

C -0.1178080145 -2.7215150887 -2.0334934035

C -1.0058791259 -3.5814603601 0.0342804740

C -1.0681470739 -3.4533306364 -2.7450673678

H 0.6029859800 -2.1227459438 -2.5871383247

C -1.9541773776 -4.3158901575 -0.6764393309

H -1.0012197205 -3.6622392290 1.1205612704

C -1.9956475962 -4.2450248853 -2.0682559326

H -1.0801128736 -3.4027516276 -3.8354256010

H -2.6668793796 -4.9410681522 -0.1362265516

H -2.7472477785 -4.8089170908 -2.6243087848

H -0.1911179550 -0.4144408611 0.5015170086

B -0.7254268333 0.6455364659 -0.0185601777

C -1.8507306328 0.0675563607 -1.0388104062

C -1.5302887570 1.4183279210 1.1720630598

C 0.4995357218 1.5018689024 -0.6786696462

C -2.0973922901 0.4854059556 -2.3437378200

C -2.7056695467 -0.9265312740 -0.5620610685

C -1.7977543744 0.9251206737 2.4414051204

C -2.1220999480 2.6470238458 0.8800023709

C 1.1295005477 1.0880592496 -1.8519333960

C 1.0911465321 2.6166423228 -0.0821627551

C -3.0690707605 -0.0983075704 -3.1549133937

F -1.4057544777 1.4879240225 -2.8871489530

C -3.6867027564 -1.5357035578 -1.3341687821

F -2.5947911816 -1.3365050054 0.7052250134

C -2.5805499117 1.5983416657 3.3788772794

F -1.2934784045 -0.2508377089 2.8425228507

C -2.9082190304 3.3568144457 1.7780306304

F -1.9253319664 3.1944732572 -0.3214209464

C 2.2466831141 1.7047052449 -2.4080250276

F 0.6811396268 0.0117658928 -2.4998721625

C 2.1901045955 3.2851888960 -0.6140073038

F 0.6699357286 3.0697773927 1.1040393956

C -3.8632554567 -1.1193139742 -2.6485832164

F -3.2524011759 0.3224826363 -4.4007230226

F -4.4485232925 -2.5024160926 -0.8378495407

C -3.1415349372 2.8219948333 3.0423003672

F -2.7952535276 1.0776673325 4.5805390728

F -3.4392473568 4.5264325127 1.4483488784

C 2.7822743951 2.8198961807 -1.7803427893

F 2.8196861023 1.2129616038 -3.4972124930

F 2.7447568279 4.2965063413 0.0436999013

F -4.7847921260 -1.6909076747 -3.4104075367

F -3.8897821103 3.4787037001 3.9158590413

F 3.8749893331 3.3999040350 -2.2498999241

**Int7**

M06-2X/Def2SVP Electronic E: -3681.523219 a.u.

M06-2X/Def2SVP Gibbs free E: -3681.137517 a.u.

M06-2X/Def2TZVPP Electronic E: -3685.205761 a.u.

C -1.7542982733 0.2975235402 3.2067358782

C -2.0092317482 0.5732954039 1.8643441320

C -3.0407601316 -0.0987640044 1.1861512029

C -3.8095008808 -1.0467325796 1.8815540441

C -3.5438039344 -1.3296413201 3.2210699553

C -2.5168552154 -0.6575425176 3.8820306493

H -0.9519147752 0.8242778657 3.7224446988

H -1.3915789901 1.3145916658 1.3468600906

H -4.6219346109 -1.5785118808 1.3798340285

H -4.1380219048 -2.0795777717 3.7455276340

H -2.3055041039 -0.8793812641 4.9298964919

Si -3.5048540135 0.3010031047 -0.5595917442

C -4.2300453058 -1.0554289025 -1.5906436885

C -3.9915194598 -2.4100855148 -1.2975653594

C -5.0019681913 -0.7260611019 -2.7190071448

C -4.5165279730 -3.4086021837 -2.1169375055

H -3.3862338023 -2.6915232231 -0.4339634824

C -5.5232942993 -1.7270711017 -3.5351461291

H -5.2021783578 0.3210542965 -2.9593315714

C -5.2810165523 -3.0679929134 -3.2328576855

H -4.3256745044 -4.4566667630 -1.8816745540

H -6.1225018058 -1.4605608229 -4.4071435292

H -5.6911930461 -3.8524446013 -3.8714487032

C -3.1453284242 3.1049913641 -0.9214506889

C -1.9760211488 2.4657602015 -1.3606673086

C -0.8150481430 3.1887543519 -1.6233697165

C -0.8122315777 4.5672519502 -1.4270468409

C -1.9677927520 5.2102675617 -0.9789926559

C -3.1337916132 4.4874139243 -0.7315804868

H 0.0842849790 2.6820593919 -1.9720912421

H 0.1021858820 5.1334344576 -1.6112912615

H -1.9627363455 6.2888710939 -0.8165737006

H -4.0364812208 4.9938008878 -0.3871941868

N -2.0572347909 1.0414176676 -1.4921239161

S -4.6286237940 2.1453548524 -0.6912978202

C -1.2565007865 0.3579444567 -2.2325220895

H 0.5097194172 -0.1178973864 -1.1027018595

B 1.3584784725 -0.2255614672 -0.2125606105

C 0.7570640016 -1.2354417728 0.9169763031

C 2.6396924107 -0.9671560202 -0.9052835182

C 1.6726535855 1.3059566951 0.2665849551

C 1.4569071409 -1.5124263733 2.0887948136

C -0.4261655297 -1.9446342728 0.7589784902

C 2.4044900226 -2.0449026535 -1.7566417093

C 3.9804104511 -0.6566126936 -0.6991309376

C 1.4526512626 1.8787250208 1.5156102951

C 2.1308629077 2.2051173090 -0.6961772193

C 0.9920151095 -2.3732879510 3.0767247172

F 2.6316506197 -0.9170550029 2.3031586558

C -0.9210384793 -2.8393283236 1.7036941134

F -1.1841122540 -1.7839356503 -0.3396302435

C 3.4136265538 -2.7670429351 -2.3845454980

F 1.1473449485 -2.4327417672 -2.0034514510

C 5.0252227514 -1.3518641991 -1.3063117486

F 4.3333923289 0.3471399776 0.1070694592

C 1.6491708837 3.2325577960 1.7942970416

F 0.9980387931 1.1526188321 2.5412916189

C 2.3384669241 3.5588443628 -0.4727042579

F 2.3155385846 1.7838127096 -1.9559407830

C -0.2149685821 -3.0394264291 2.8812024963

F 1.6813778087 -2.5742025386 4.1935288332

F -2.0623557763 -3.4921109269 1.4955476908

C 4.7389173146 -2.4147955080 -2.1537134442

F 3.1289965039 -3.7828283571 -3.1924246778

F 6.2890617508 -1.0125036273 -1.0797918470

C 2.0912389435 4.0824468706 0.7913592734

F 1.3988599886 3.7115911958 3.0068432866

F 2.6967583861 4.3659888302 -1.4665701300

F -0.6877542920 -3.8559533890 3.8122221604

F 5.7206773200 -3.0880383395 -2.7383980014

F 2.2506984556 5.3778054083 1.0220216290

H -0.5038110362 0.8581307262 -2.8478906834

H -1.3833710769 -0.7236947215 -2.3133560067

**Product**

M06-2X/Def2SVP Electronic E: -1475.653229 a.u.

M06-2X/Def2SVP Gibbs free E: -1475.391004 a.u.

M06-2X/Def2TZVPP Electronic E: -1476.754156 a.u.

C 2.4564572108 2.9972033651 -1.6714793426

C 1.4142715898 2.2072592296 -2.1569541661

C 0.7651462030 1.2835330409 -1.3219265058

C 1.1879757396 1.1707788559 0.0123363126

C 2.2252514361 1.9638398556 0.5017650206

C 2.8600649800 2.8774272711 -0.3410205104

H 2.9541170071 3.7092990552 -2.3322079722

H 1.1049781117 2.3140278429 -3.2004506433

H 0.6993938710 0.4514707402 0.6757714750

H 2.5411702926 1.8681214264 1.5421194662

H 3.6733579948 3.4977930382 0.0403343255

Si -0.5886296408 0.1584900361 -1.9533349961

H -2.1344597265 1.4642327967 -0.0179721560

C -1.5864873452 1.0238871075 -3.2837516876

C -1.8377670343 2.4046708716 -3.2038287497

C -2.1663923592 0.2982544998 -4.3363256204

C -2.6474151297 3.0395501634 -4.1458378916

H -1.3946792257 2.9980601660 -2.3987393778

C -2.9710084888 0.9319370020 -5.2828153333

H -1.9809900297 -0.7759150276 -4.4184036995

C -3.2129450676 2.3028807741 -5.1871948349

H -2.8344234973 4.1121632453 -4.0690749588

H -3.4102794490 0.3542925250 -6.0981306936

H -3.8430263160 2.7992306147 -5.9277222103

C -0.8641946643 -2.6321140310 -1.3429418772

C -1.6269283661 -1.8308430467 -0.4589734347

C -2.4029361328 -2.4659753597 0.5202891657

C -2.4064456613 -3.8595688751 0.6197155824

C -1.6466617031 -4.6386706799 -0.2484418662

C -0.8732825632 -4.0176240103 -1.2346125290

C -2.3326706846 0.4133221090 0.2373573695

H -3.0048755405 -1.8710360200 1.2071802494

H -3.0161623192 -4.3355141480 1.3898799644

H -1.6519222807 -5.7263101997 -0.1676116544

H -0.2754999766 -4.6148652166 -1.9260304146

H -2.0711991013 0.2708507794 1.2989502968

N -1.5517640286 -0.4557048756 -0.6211375656

S 0.0973666153 -1.7948465485 -2.5898011063

H -3.4146303096 0.2349533269 0.1199357499

**TS2/3’**

M06-2X/Def2SVP Electronic E: -3681.478616 a.u.

M06-2X/Def2SVP Gibbs free E: -3681.090905 a.u.

M06-2X/Def2TZVPP Electronic E: -3685.164690 a.u.

C -0.2873278258 3.8191023773 2.7170497404

C 0.6695967522 2.9353817156 2.2236222528

C 0.5294515811 1.5516523531 2.4452900898

C -0.5761357319 1.0720527657 3.1752905805

C -1.5123668592 1.9639321012 3.6866626418

C -1.3704628820 3.3339894409 3.4519814933

H -0.1830174679 4.8896085203 2.5346783609

H 1.5328446891 3.3259981329 1.6790487180

H -0.7199642828 -0.0005377318 3.3239687479

H -2.3739692923 1.5889831072 4.2405409566

H -2.1220949515 4.0283869339 3.8324277194

Si 1.8903975930 0.4269426793 1.9994565966

C 2.2570451148 -1.0718872679 2.9747048032

C 3.0564135389 -2.0862337203 2.4094192126

C 1.7685263460 -1.2219344352 4.2858723513

C 3.3500567178 -3.2295133320 3.1437924977

H 3.4466178086 -1.9813270818 1.3936796866

C 2.0724753871 -2.3686031058 5.0156316098

H 1.1619317876 -0.4399572398 4.7475396045

C 2.8567828135 -3.3702149452 4.4434055003

H 3.9627394716 -4.0156751464 2.7009069526

H 1.6962904245 -2.4799092745 6.0333532094

H 3.0886679712 -4.2701559769 5.0157264366

C 4.2810539047 1.0187706625 -1.0104391337

C 3.1459825794 1.4427483055 -0.2970706526

C 2.1069353168 2.0921213580 -0.9568349674

C 2.2202029026 2.3237889214 -2.3313610343

C 3.3493050244 1.9063622157 -3.0349746437

C 4.3894458118 1.2448029376 -2.3777321122

C 4.5687226169 0.8024414887 1.4686648252

H 1.2052887976 2.4018137150 -0.4244429906

H 1.4070949174 2.8322492143 -2.8486787467

H 3.4210164084 2.0889730053 -4.1082415189

H 5.2669677531 0.8993939234 -2.9262699853

H 4.6538570628 0.1148839964 2.3185975630

N 3.1776156009 1.0366306573 1.0696116353

S 5.4145857543 0.1132759436 0.0007458221

H -0.5066769139 -0.5151133202 0.7698261416

H 5.0362894483 1.7636966376 1.7300657444

B -1.1188643845 -0.3793247972 -0.2921148912

C -2.1811221102 -1.6218470775 -0.3710521952

C -1.8200701491 1.0934134233 -0.2145653204

C -0.0530757054 -0.5928010689 -1.5061197234

C -3.5140324347 -1.5478865090 -0.7634040163

C -1.7257286050 -2.9027912855 -0.0628014891

C -2.7330544277 1.3290172837 0.8136301881

C -1.5559609241 2.2075334177 -1.0057905230

C -0.4080044056 -0.4660486747 -2.8460143147

C 1.2510473321 -0.9998032440 -1.2837326288

C -4.3539897309 -2.6586183546 -0.8268985574

F -4.0638440852 -0.3791958071 -1.1037128147

C -2.5259078541 -4.0394060727 -0.1130735286

F -0.4524357934 -3.0829553430 0.3049371598

C -3.3272359312 2.5577431005 1.0655157626

F -3.0604974882 0.3325174024 1.6434038614

C -2.1278253581 3.4630800766 -0.7951896318

F -0.6999256661 2.1436056016 -2.0329603583

C 0.4809539552 -0.6743177434 -3.8944840350

F -1.6538795424 -0.1064858786 -3.1592621880

C 2.1769657631 -1.2528078957 -2.2907314163

F 1.7083140448 -1.1500046163 -0.0165976090

C -3.8549781604 -3.9134639911 -0.5020764822

F -5.6217778893 -2.5309623685 -1.2030133233

F -2.0376618496 -5.2359275445 0.1971790993

C -3.0163127606 3.6429516485 0.2542340918

F -4.1370448087 2.7212042807 2.1069778009

F -1.8108274404 4.4872696278 -1.5808901092

C 1.7868897747 -1.0668034212 -3.6097869765

F 0.1076610642 -0.5041121174 -5.1570782834

F 3.4138199380 -1.6436791341 -2.0092559346

F -4.6386162915 -4.9823666035 -0.5618299845

F -3.5457718149 4.8349441188 0.4974924386

F 2.6579000500 -1.2481939651 -4.5926777770

**Int3’**

M06-2X/Def2SVP Electronic E: -3681.486728 a.u.

M06-2X/Def2SVP Gibbs free E: -3681.099918 a.u.

M06-2X/Def2TZVPP Electronic E: -3685.173638 a.u.

C 0.0058226861 -4.1411535932 1.2305482443

C -0.9982041560 -3.1985973117 1.0160972694

C -1.0133411748 -2.0032367740 1.7552172141

C -0.0209587490 -1.7774763946 2.7286169338

C 0.9704866558 -2.7279874065 2.9495650294

C 0.9862394127 -3.9034865124 2.1946280441

H 0.0206893412 -5.0635001299 0.6475337104

H -1.7783078288 -3.4030372447 0.2787055178

H -0.0190028091 -0.8547886800 3.3145784397

H 1.7457112782 -2.5445177544 3.6944601410

H 1.7778829430 -4.6384200883 2.3561536159

Si -2.3892435473 -0.8108678937 1.5954284680

C -3.0922326725 -0.0767818287 3.1235401356

C -3.7861756889 1.1461624703 3.0609118074

C -2.9457139959 -0.7261800017 4.3598466881

C -4.3218600539 1.7048401636 4.2170110615

H -3.9105700470 1.6638536314 2.1054657753

C -3.4894180872 -0.1638736482 5.5143791022

H -2.4073689575 -1.6748058385 4.4269381036

C -4.1735722867 1.0487614053 5.4417498298

H -4.8551840313 2.6550433224 4.1647700005

H -3.3755070353 -0.6732662783 6.4722928897

H -4.5950946371 1.4894853705 6.3469305683

C -4.4018232089 -0.4367649930 -1.7830745912

C -3.3147706156 -0.9864997069 -1.0792167857

C -2.1596472893 -1.3587766566 -1.7587057501

C -2.0909569379 -1.1585630859 -3.1413588005

C -3.1625702348 -0.5977286262 -3.8342912167

C -4.3297570689 -0.2339687701 -3.1566070235

C -4.9643404216 -1.0016040172 0.6009874530

H -1.3011238729 -1.7738670691 -1.2299362466

H -1.1814849936 -1.4450597717 -3.6700489321

H -3.0949442368 -0.4424784779 -4.9119064961

H -5.1716034596 0.2056356976 -3.6934474114

H -5.2090635647 -0.5862964891 1.5847438932

N -3.5223377233 -0.9974116819 0.3304682247

S -5.7385288883 0.0100457370 -0.7157151813

H 1.0351533480 0.3273692453 1.3125571219

H -5.3529368629 -2.0285501809 0.5285328509

B 1.4257652352 0.3959626694 0.1615874199

C 2.6060352557 1.5289523044 0.1270555827

C 1.9584057856 -1.0903845318 -0.2499350140

C 0.1850080795 0.9741361847 -0.7422078151

C 3.8028307802 1.4486621607 -0.5789622111

C 2.4183387785 2.7101221324 0.8424625276

C 2.8620274703 -1.7129733366 0.6106372813

C 1.6178387627 -1.8443820086 -1.3677528886

C 0.3528981499 1.3804297562 -2.0685988892

C -1.0773815722 1.2227150463 -0.2577785021

C 4.7690482650 2.4532857969 -0.5579159142

F 4.0844726415 0.3812240749 -1.3297200050

C 3.3521702020 3.7400427485 0.8906097996

F 1.2856565309 2.8974966912 1.5287037164

C 3.3915200311 -2.9797562584 0.3979345699

F 3.2520028540 -1.0872733949 1.7249627910

C 2.1130456151 -3.1226394312 -1.6213107664

F 0.7689051118 -1.3670991495 -2.2882159278

C -0.6582646058 1.9559362442 -2.8287199725

F 1.5303428744 1.2022631238 -2.6606823050

C -2.1265175403 1.8300690904 -0.9362136988

F -1.4176670213 0.8121483216 1.0515743363

C 4.5405869923 3.6070425687 0.1811219115

F 5.8987586972 2.3239573965 -1.2449770883

F 3.1211671814 4.8429447755 1.5949260904

C 3.0068459685 -3.6970440900 -0.7284966688

F 4.2065721388 -3.5351210823 1.2893448365

F 1.7301644327 -3.7942805058 -2.7023819252

C -1.9127023833 2.1803851707 -2.2597256117

F -0.4568478048 2.2825743751 -4.0947364890

F -3.3053789790 2.0128738042 -0.3617132156

F 5.4469735107 4.5750676449 0.2080458827

F 3.4715038056 -4.9226738038 -0.9342795724

F -2.8861807717 2.7101273737 -2.9797409629

**TS3’/4’**

M06-2X/Def2SVP Electronic E: -3681.452441 a.u.

M06-2X/Def2SVP Gibbs free E: -3681.066710 a.u.

M06-2X/Def2TZVPP Electronic E: -3685.138843 a.u.

C 1.5211431703 3.7782582307 1.6402478766

C 2.3082346987 2.8431859130 0.9772304768

C 2.1843454365 1.4746003877 1.2893514383

C 1.2512628691 1.0604318544 2.2582662678

C 0.4474309565 2.0022716641 2.8959030078

C 0.5897209891 3.3567949891 2.5937802151

H 1.6183616415 4.8384734903 1.3997427236

H 3.0324991778 3.1814440691 0.2354503972

H 1.1423605107 0.0023706740 2.5084875102

H -0.2944277391 1.6745997246 3.6257761932

H -0.0360693599 4.0940441551 3.1011022823

Si 3.3657043773 0.2536735805 0.6405797840

C 4.0090365432 -0.9421922276 1.8843160638

C 4.0144731671 -2.3392169077 1.7413032226

C 4.4785007545 -0.3676125279 3.0816335586

C 4.5020551057 -3.1437657755 2.7677823774

H 3.6098958090 -2.8113130249 0.8444003232

C 4.9808255916 -1.1785174455 4.0967904641

H 4.4552717089 0.7155828837 3.2288688034

C 4.9916538676 -2.5644455899 3.9392286738

H 4.4908251605 -4.2284786622 2.6543084610

H 5.3536021586 -0.7260735778 5.0166840027

H 5.3736693066 -3.1991946389 4.7406000648

C 2.8247324363 0.0171712482 -2.3918000874

C 3.4817837583 1.1273295587 -1.8273931381

C 3.2537348115 2.3990484693 -2.3253025144

C 2.3517946207 2.5406617185 -3.3874384976

C 1.7036256267 1.4367911189 -3.9366038839

C 1.9372212423 0.1461888371 -3.4458001709

C 4.9192898844 -0.5317684130 -1.1710321047

H 3.7771685193 3.2600840140 -1.9083570583

H 2.1594798751 3.5349734063 -3.7919726014

H 1.0103954971 1.5720833792 -4.7676171658

H 1.4409554615 -0.7194562118 -3.8856605756

H 5.4984175222 -1.0423938224 -0.3922752267

N 4.3503857354 0.7427144530 -0.7348587951

S 3.3470181060 -1.4590175978 -1.5305196673

H -1.3647246845 -0.3716592060 1.8367526669

H 5.5047178023 -0.4287234422 -2.0943945485

B -1.7316009268 -0.2277604052 0.6789570552

C -3.2573619846 -0.8248927995 0.5738015063

C -1.6099267875 1.3650016820 0.3338273593

C -0.8316679124 -1.1897521120 -0.2899710897

C -4.3361557685 -0.2625642837 -0.1026965570

C -3.5172813607 -2.0592946718 1.1676960191

C -2.4726691232 2.2669746556 0.9565021597

C -0.6332170864 1.9583314224 -0.4609040703

C -1.0767191581 -1.2203206106 -1.6616342391

C 0.1309822616 -2.0832403747 0.1613302455

C -5.5960987785 -0.8557426349 -0.1626946346

F -4.2146479896 0.9053000200 -0.7397477183

C -4.7568075599 -2.6904525063 1.1333918355

F -2.5394127829 -2.7039883240 1.8131649319

C -2.4086048993 3.6459326535 0.7974162452

F -3.4320848019 1.8092688662 1.7672061455

C -0.5299396697 3.3355606107 -0.6515319693

F 0.3020041326 1.2237078569 -1.0813127918

C -0.4302284562 -2.0779517508 -2.5411452520

F -1.9690977470 -0.3767243753 -2.1835906194

C 0.7909546089 -2.9774367968 -0.6823020110

F 0.4945282683 -2.1201469110 1.4487987888

C -5.8066607640 -2.0808447271 0.4565571204

F -6.5939663631 -0.2678613927 -0.8153877578

F -4.9449302592 -3.8659058409 1.7253573691

C -1.4204133427 4.1870526578 -0.0172400139

F -3.2583012535 4.4492677540 1.4295882625

F 0.4628070424 3.8481698054 -1.3757889565

C 0.5267203932 -2.9579698324 -2.0447503954

F -0.6574587835 -2.0292925163 -3.8504725710

F 1.7370475717 -3.7892719768 -0.2127444952

F -6.9981191813 -2.6634386889 0.4031543136

F -1.2986014699 5.5035549602 -0.1480927073

F 1.2047398153 -3.7428601615 -2.8717703276

**Int4’**

M06-2X/Def2SVP Electronic E: -3681.453432 a.u.

M06-2X/Def2SVP Gibbs free E: -3681.068748 a.u.

M06-2X/Def2TZVPP Electronic E: -3685.142102 a.u.

C -1.7347286156 3.6492633507 -1.7048867446

C -2.5203056852 2.7430147181 -0.9986750314

C -2.3788927933 1.3620188579 -1.2312172569

C -1.4303574944 0.9057058312 -2.1643924231

C -0.6314996139 1.8193163401 -2.8485292425

C -0.7925080854 3.1873285495 -2.6272271957

H -1.8466421515 4.7199724554 -1.5246096379

H -3.2562785177 3.1159238168 -0.2847003973

H -1.3069183049 -0.1632742017 -2.3571621362

H 0.1191476571 1.4586392121 -3.5535532331

H -0.1731089461 3.9018985909 -3.1733109877

Si -3.5773699075 0.1587352645 -0.5600434760

C -4.2571604014 -1.0352424203 -1.7928034426

C -4.0473823699 -2.4213638094 -1.7269838049

C -4.9722143420 -0.4947115039 -2.8769179750

C -4.5575586755 -3.2538901147 -2.7214648519

H -3.4652993637 -2.8631925102 -0.9151384863

C -5.4914491112 -1.3334240257 -3.8603045046

H -5.1265240049 0.5847598662 -2.9609413544

C -5.2837276358 -2.7113495215 -3.7816748775

H -4.3811958339 -4.3291136640 -2.6689184495

H -6.0524286952 -0.9092945811 -4.6943306341

H -5.6840349097 -3.3662411200 -4.5574784502

C -2.9595479933 0.2721239489 2.2879678475

C -3.8253721921 1.2537073963 1.7686910310

C -3.7698233315 2.5480494371 2.2567249971

C -2.8339710559 2.8354336038 3.2589867453

C -1.9825814407 1.8529810078 3.7602686687

C -2.0344761134 0.5383392180 3.2795154143

C -5.0506566355 -0.5876922601 1.2249725004

H -4.4457007754 3.3115837771 1.8700206515

H -2.7732608332 3.8502261002 3.6541331904

H -1.2643131198 2.1046952667 4.5411710014

H -1.3635547493 -0.2300518601 3.6657992900

H -5.6029449521 -1.2005867593 0.5025585039

N -4.6867260668 0.7340339993 0.7290151723

S -3.2959153517 -1.2713944294 1.4279918783

H 1.3450627262 -0.4143208401 -1.8801374572

H -5.5389100043 -0.5742932691 2.2078553109

B 1.7028950848 -0.2450000433 -0.7232167262

C 3.2406976299 -0.8053246342 -0.5966839641

C 1.5438105542 1.3516826900 -0.4102244911

C 0.8106609246 -1.2074992621 0.2527685455

C 4.3054416784 -0.1991269099 0.0639356858

C 3.5298010404 -2.0508983515 -1.1530858273

C 2.3741559675 2.2612657108 -1.0647100974

C 0.5622442358 1.9398560348 0.3815229303

C 1.0074433733 -1.1809744422 1.6315188619

C -0.1117981407 -2.1445862268 -0.1976051904

C 5.5779054865 -0.7628106374 0.1456089030

F 4.1576400567 0.9848379861 0.6649539424

C 4.7824467445 -2.6533905548 -1.0961082054

F 2.5689978498 -2.7358445491 -1.7823202567

C 2.2701132529 3.6417739221 -0.9419165295

F 3.3401243441 1.8094340985 -1.8710075581

C 0.4209311900 3.3171214273 0.5412020281

F -0.3443612941 1.1979125290 1.0344597238

C 0.3419290056 -2.0122145201 2.5236219903

F 1.8606460234 -0.2971945899 2.1524050039

C -0.7863710720 -3.0140273739 0.6601551577

F -0.4173283893 -2.2508009414 -1.4945687426

C 5.8169834720 -2.0012320472 -0.4353239703

F 6.5602000515 -0.1331743945 0.7827647864

F 4.9977284629 -3.8415362110 -1.6522290865

C 1.2771742493 4.1767485503 -0.1283243015

F 3.0902912196 4.4524324293 -1.6032892713

F -0.5701063271 3.8144151663 1.2778418842

C -0.5744821617 -2.9344568822 2.0296716218

F 0.5173589796 -1.8982059272 3.8372388850

F -1.7041104824 -3.8613148158 0.1942993934

F 7.0202779544 -2.5565954883 -0.3617408593

F 1.1233575527 5.4924266660 -0.0266564528

F -1.2681248266 -3.7020891252 2.8590120346
